# Identification of Targets of CD8^+^ T Cell Responses to Malaria Liver Stages by Genome-wide Epitope Profiling

**DOI:** 10.1371/journal.ppat.1003303

**Published:** 2013-05-09

**Authors:** Julius Clemence R. Hafalla, Karolis Bauza, Johannes Friesen, Gloria Gonzalez-Aseguinolaza, Adrian V. S. Hill, Kai Matuschewski

**Affiliations:** 1 Department of Immunology and Infection, Faculty of Infectious and Tropical Diseases, London School of Hygiene and Tropical Medicine, London, United Kingdom; 2 The Jenner Institute, University of Oxford, Old Road Campus Research Building, Oxford, United Kingdom; 3 Parasitology Unit, Max Planck Institute for Infection Biology, Berlin, Germany; 4 Department of Gene Therapy and Hepatology, Center for Investigation in Applied Medicine (CIMA), University of Navarra, Pamplona, Spain; 5 Institute of Biology, Humboldt University, Berlin, Germany; Weill Medical College of Cornell University, United States of America

## Abstract

CD8^+^ T cells mediate immunity against *Plasmodium* liver stages. However, the paucity of parasite-specific epitopes of CD8^+^ T cells has limited our current understanding of the mechanisms influencing the generation, maintenance and efficiency of these responses. To identify antigenic epitopes in a stringent murine malaria immunisation model, we performed a systematic profiling of H^2b^-restricted peptides predicted from genome-wide analysis. We describe the identification of *Plasmodium berghei* (*Pb*) sporozoite-specific gene 20 (S20)- and thrombospondin-related adhesive protein (TRAP)-derived peptides, termed *Pb*S20_318_ and *Pb*TRAP_130_ respectively, as targets of CD8^+^ T cells from C57BL/6 mice vaccinated by whole parasite strategies known to protect against sporozoite challenge. While both *Pb*S20_318_ and *Pb*TRAP_130_ elicit effector and effector memory phenotypes in both the spleens and livers of immunised mice, only *Pb*TRAP_130_-specific CD8^+^ T cells exhibit *in vivo* cytotoxicity. Moreover, *Pb*TRAP_130_-specific, but not *Pb*S20_318_-specific, CD8^+^ T cells significantly contribute to inhibition of parasite development. Prime/boost vaccination with *Pb*TRAP demonstrates CD8^+^ T cell-dependent efficacy against sporozoite challenge. We conclude that *Pb*TRAP is an immunodominant antigen during liver-stage infection. Together, our results underscore the presence of CD8^+^ T cells with divergent potencies against distinct *Plasmodium* liver-stage epitopes. Our identification of antigen-specific CD8^+^ T cells will allow interrogation of the development of immune responses against malaria liver stages.

## Author Summary

Vaccination against malaria is feasible, as demonstrated with radiation-attenuated sporozoite vaccine, which protects experimental animals and humans by targeting the clinically silent liver stages. Potent protection largely depends on CD8^+^ T cells, a type of white blood cell that is tailor-made to kill obligate intracellular pathogens. Malaria-infected cells display fragments of parasite proteins, which are then recognised and targeted by CD8^+^ T cells. How CD8^+^ T cells are activated following immunisation and how they execute protective functions are key considerations for vaccination. However, characterisation of CD8^+^ T cells is hampered by the lack of identified malaria protein targets. Of concern, the circumsporozoite protein, which is the basis of the most advanced malaria vaccine candidate (RTS,S), is not an essential target of CD8^+^ T cells induced by attenuated sporozoites in several mouse strains. In this study, we have made considerable advances by identifying for the first time, fragments of malaria proteins that are targeted by CD8^+^ T cells generated by vaccination in a relevant mouse strain, C57BL/6. Notably, CD8^+^ T cells against one of the target proteins elicit partial protection against infection. Our study exemplifies how immunisation by complex pathogens can be dissected to identify distinct antigens for subunit vaccine development.

## Introduction

Malaria is responsible for an estimated 250 million episodes of clinical disease and 600,00 to 1.2 million deaths each year [1], [2]. Notwithstanding recent reductions in the burden of malaria in some endemic areas, sustained control, elimination or eradication of the disease will require a highly efficacious vaccine that prevents malaria transmission as well as reducing the burden of disease. As a benchmark in malaria vaccination, multiple immunisations of γ-radiation-attenuated *Plasmodium* sporozoites (γ-Spz) can protect both mice and humans against sporozoite challenge [3], [4]. The elicited protection targets the development of liver stages and completely prevents blood stage infection, resulting in sterile immunity. This experimental vaccine approach has now been replicated using other whole sporozoite immunisation strategies that include infection under drug cover and genetically arrested parasites [5]–[8]. Naturally acquired pre-erythrocytic immunity is likely multifactorial [9], involving both antibodies and T cells. However, CD8^+^ T cells are the prime mediators of protection after γ-Spz vaccination in mice [10], [11], and interferon (IFN)-γ is a signature of effector function [12].

How CD8^+^ T cells are primed, modulated, and maintained following immunisation, and how these cells execute protective functions, are key considerations for vaccine design and can only be addressed with antigen-specific tools. The circumsporozoite protein (CSP), the major surface protein of the sporozoite, has been at the forefront of vaccination studies for more 20 years – being the basis of RTS,S, the most advanced malaria vaccine to date [13]. Furthermore, CSP-specific responses have been the standard in measuring cellular responses to malaria liver stages in fundamental immunological studies in mice [14], [15].

Murine models of sporozoite immunisation have largely focused on two strains, BALB/c and C57BL/6 (B6). Immunisation with *Plasmodium berghei* (*Pb*) or *P. yoelii* (*Py*) γ-Spz induces highly protective, H2^d^-restricted CD8^+^ T cell responses to defined CSP epitopes in BALB/c mice [16], [17]. However, protection can also be obtained in the absence of *Py*CSP-specific T cells: (a) *Py*CSP-transgenic BALB/c mice - that are tolerant to CSP - can be completely protected by *Py* γ-Spz immunisation [18] and (b) there is cross-species immunity to sporozoites despite lack of cross-reactivity of the CSP-derived CD8^+^ T cell epitopes [19]. These data highlight the importance of non-CSP antigens in generation of protective immunity to liver stages. However, the paucity of liver-stage specific antigens for CD8^+^ T cells, and the limited availability of gene-targeted mice on the BALB/c background, has limited both the evaluation of subunit vaccine candidates in murine malaria models and the characterisation of the mechanisms underlying CD8^+^ T cell mediated protection.

In contrast to the ease of inducing protective immunity in BALB/c mice, B6 mice expressing H-2^b^ can only be protected against *Pb* (or *Py*) infection by multiple rounds of γ-Spz immunisation. Most importantly, protection is entirely independent of CSP-specific CD8^+^ T cells [18]. Indeed, the CSP seems to contain no naturally processed and presented H-2^b^-restricted epitopes. We propose, therefore, that the *Pb*-B6 model is a more relevant model of liver stage immunity than the BALB/c model. It more closely resembles the situation in humans, where CD8^+^ T cell responses to the CSP are infrequent [9]. These immune-epidemiological findings in malaria-endemic areas are reflected by the fact that multiple immunisations are needed to elicit sterilising immunity [20] Moreover, B6 mice are particularly attractive for immunological studies due to the availability of a large collection of sub-strains with targeted gene deletions.

In order to develop a *Pb*-B6 model of antigen-specific CD8^+^ T cell-mediated anti-liver stage immunity, we employed an unbiased genome-wide approach for screening H-2^b^ (K^b^ and D^b^) restricted *Pb*-derived peptides that are recognised by CD8^+^ T cells from B6 mice immunised with whole sporozoite immunisation strategies known to induce protection. Our results identify two novel liver stage immunogenic targets of effector CD8^+^ T cells in immunised B6 mice. Of these two, CD8^+^ T cell responses to *PbTRAP* confers partial efficacy against sporozoite challenge *in vivo*. Considering that *P. falciparum* TRAP (*Pf*TRAP) is a major target of human malaria vaccine development, our results emphasize the translational relevance of the *Pb*-B6 model.

## Results

### Systematic profiling of CD8^+^ T cell epitopes in sporozoite-immune B6 mice

The data and tools available at the Immune Epitope Database and Analysis Resource (www.iedb.org) were used for the identification of putative CD8^+^ T cell epitopes [21]. Systematic epitope profiling has previously identified previously unrecognized CD8^+^ T cell responses to a number of viral infections including vaccinia, dengue and herpes viruses [22]–[24]. To assemble a genome-wide peptide library, *Pb* open-reading frames, based on published sporozoite and liver stage transcriptomic and proteomic data [25]–[29], were scanned *in silico* using artificial neural network methods [30] for major histocompatibility complex (MHC) Class I H2-K^b^ and D^b^ restricted peptides. In addition, predictions were performed using stabilised matrix methods [31] on the entire *Pb* draft genome [32]. Finally, *Pb* orthologs of *Pf* proteins that were reported to be antigenic for either human antibodies or T cells from individuals immunised by irradiated *Pf* sporozoites (*Pf* γ-Spz) [33], [34] were also analysed.

**Figure 1:**
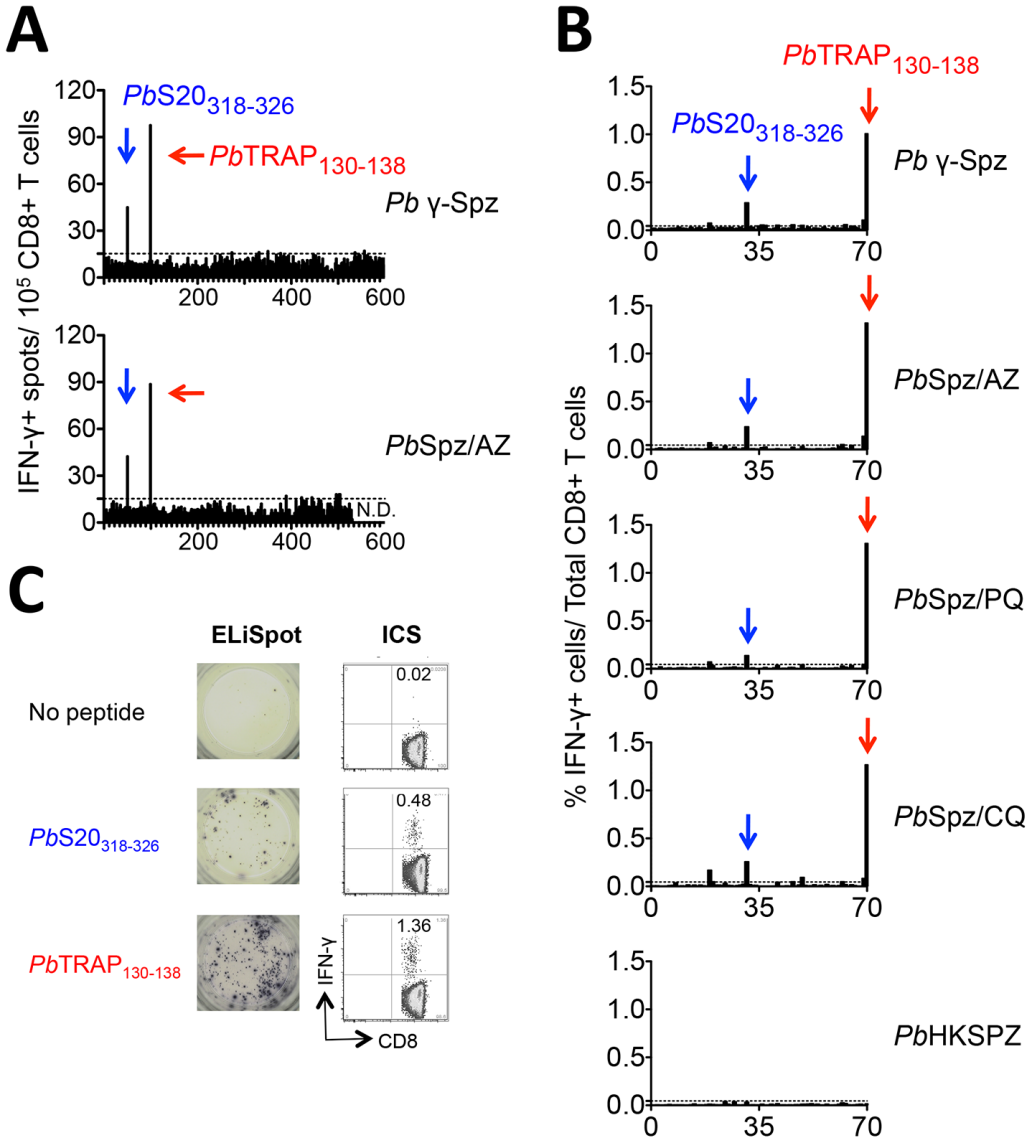
Systematic profiling of CD8^+^ T cell epitopes in sporozoite-immune B6 mice: Identification of *Pb*S20_318_ (*Pb*S20_318–325_) and *Pb*TRAP_130_ (*Pb*TRAP_130–138_) as targets of CD8^+^ T cell responses. (**A**) 600 peptides, which were predicted from *Pb* sequences to bind H-2^b^ molecules, were screened by ELISpot for their ability to induce IFN-γ secretion in CD8^+^ T cells isolated and purified from mice immunised with *Pb* γ-Spz (top) and *Pb*Spz/AZ (bottom). (**B**) The same peptides were used to stimulate spleen cells, and IFN-γ secretion from CD8^+^ T cells was measured by ICS. Data shown is based on a sub-set of 70 peptides. From top: Spleen cells were isolated from mice immunised twice with *Pb* γ-Spz, *Pb*Spz/AZ, *Pb*Spz/PQ, *Pb*Spz/CQ and *Pb*HKSpz. For (A) and (B), dotted lines represent cut-offs calculated using a mixture model. (**C**) Representative ELISpot wells (left) and ICS flow cytometry plots (right) of CD8^+^ T cells isolated from mice immunized twice with *Pb* γ-Spz. Frequencies of IFN-γ^+^ cells/total CD8^+^ T cells are indicated.

**Figure 2:**
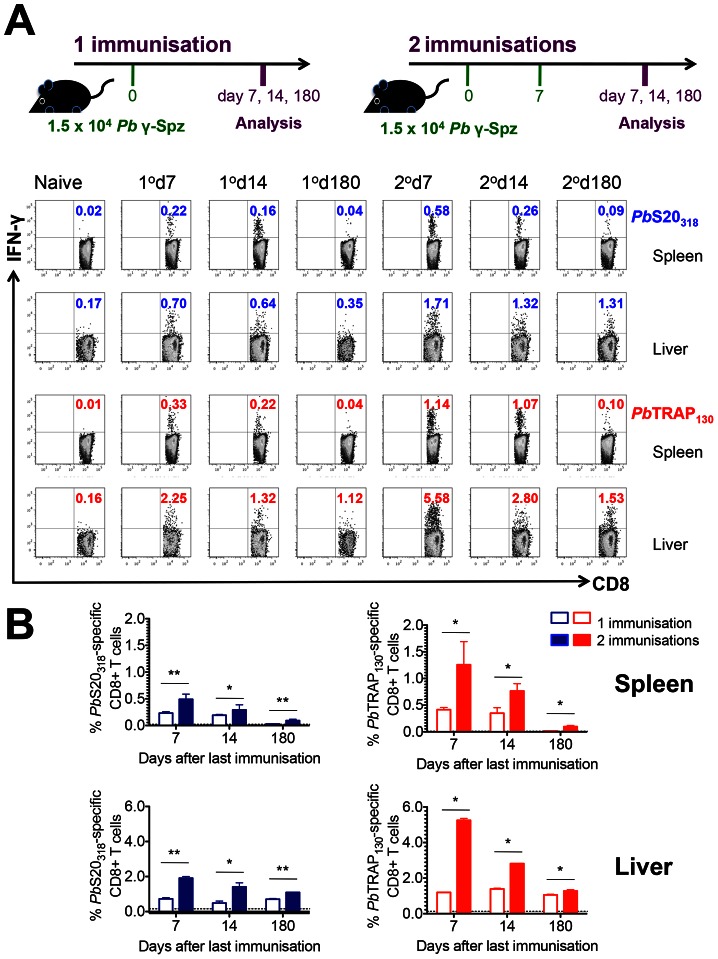
Kinetics of *Pb*S20_318_ and *Pb*TRAP_130_-specific CD8^+^ T cell responses following immunisation with *Pb* γ-Spz. (**A**) B6 mice were immunised either once or twice with *Pb* γ-Spz as shown in the schematic diagram. On days 7, 14 and 180 after the last immunisation, *Pb*S20_318_ and *Pb*TRAP_130_-specific CD8^+^ T cell responses were quantified in the spleens and the livers by peptide stimulation followed by ICS. Representative flow cytometry plots showing IFN-γ-secretion by CD8^+^ T cells in the spleens and the livers of *Pb* γ-Spz-immunised mice. (**B**) Data in (A) presented as bar graphs: white squares = 1° immunisation, black squares = 2° immunisation (blue for *Pb*S20_318_ and red for *Pb*TRAP_130_), differences between 1° vs 2° immunisations: **p<0.01 and *p<0.05, Mann-Whitney test. Dotted lines represent baseline responses based on peptide stimulation of spleens from naïve mice. Experiments were performed at least 5 times with 3–5 mice per group. Frequencies of epitope-specific CD8^+^ T cells were also compared among the different time points and were found to be statistically different (p<0.05) using the Kruskal Wallis test.

From this *in silico* analysis, 600 unique peptide sequences (288 8-mers, 311 9-mers and one 10-mer), which correspond to >350 *Pb* antigens, were identified and subsequently produced by solid phase synthesis (**Table S1**: Summary of datasets and **Table S2**: Complete list of peptides). Individual peptides were tested and CD8^+^ T cell-derived IFN-γ was quantified by two complementary read-outs: (1) an enzyme-linked immunospot (ELiSpot) assay [35] (**Figure 1A,C**), and (2) direct peptide-stimulation followed by intracellular cytokine staining (ICS) (**Figure 1B,C**). Animals received two immunisation doses of one of four whole sporozoite vaccination strategies: (i) *Pb* γ-Spz [3] and live sporozoites (*Pb*Spz) given concomitantly with anti-malaria drugs (ii) azithromycin (AZ) [8], (iii) primaquine (PQ) [7] or (iv) chloroquine (CQ) [6], [36]. As negative controls, CD8^+^ T cells were isolated from mice immunised with heat-killed sporozoites (*Pb*HKSpz), known to elicit sub-optimal T cell responses [**37**], [**38**], and naïve mice.

### Liver stage antigen epitopes *Pb*S20_318_ and *Pb*TRAP_130_ correlate with CD8^+^ T cell-mediated protection

Two peptides consistently elicited robust IFN-γ responses in a proportion of CD8^+^ T cells isolated across all four whole sporozoite vaccine strategies (**Figure 1A,B**) but not from *Pb*HKSpz (**Figure 1B**) and naïve mice (data not shown). Several other peptides were weakly reactive during initial screens but were not confirmed upon re-screening.

The first peptide, VNYSFLYLF, contains motifs for K^b^ and is derived from amino acids 318–325 of the *Pb*S20 protein [*Pb*S20_318_ (or *Pb*S20_318–325_)] (PBANKA_142920; gi: 40950503), an uncharacterised protein that is conserved in *Pf* (**Figure S1**). S20 was first identified as a sporozoite-specific gene in *Py* [26]. *Pb*S20_318_ is located within a galactose oxidase (central domain) superfamily motif of the protein (**Figure S1**).

The second peptide, SALLNVDNL, is restricted for D^b^ and is derived from amino acids 130–138 of the *Pb*TRAP [*Pb*TRAP_130_ (or *Pb*TRAP_130–138_)] (PBANKA_134980; gi: 1813523) [39], also known as sporozoite surface protein 2 [40] or sporozoite gene 8 (S8) [26] (**Figure S1**). Conserved in *Pf* (**Figure S1**), TRAP is a secreted transmembrane protein of sporozoites that plays a vital role in parasite motility and invasion of hepatocytes [41]. *Pb*TRAP_130_ is located within the von Willebrand factor type A domain (**Figure S1A**), the key motif for parasite locomotion and target cell entry [42]. Reactive CD8^+^ T cells to *Pb*TRAP_130_ are considerably more abundant than those reactive to *Pb*S20_318_ (**Figure 1A–C**). The identification of a *Pb*TRAP-derived peptide as a target of CD8^+^ T cells in B6 mice is of interest since *Pf*TRAP has been a major target for malaria vaccine development in humans [43]–[46]. Thus far, no CD8^+^ T cell epitope in TRAP has been identified in a murine model, meaning that fundamental studies on the induction, differentiation and long-term persistence of protective TRAP-specific cells following parasite immunisation could not be carried out yet.

### *Pb*S20_318_- and *Pb*TRAP_130_-specific CD8^+^ T cells persist to long-term memory

**Figure 3:**
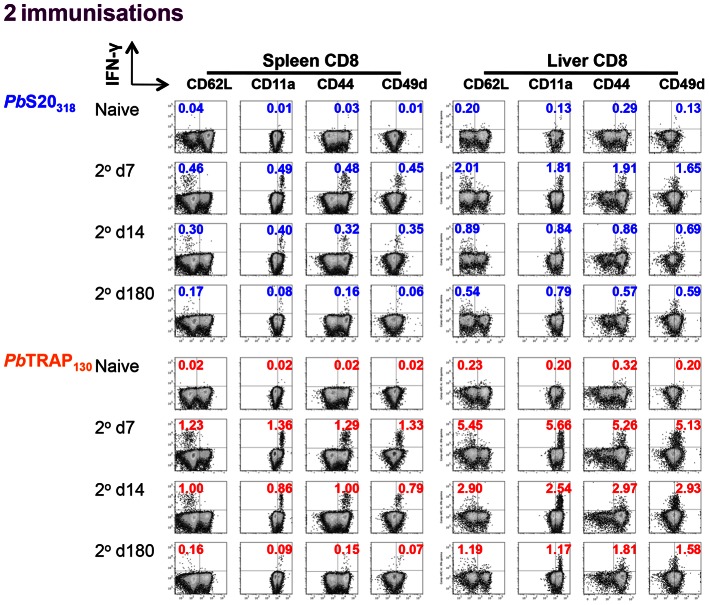
Phenotyping *Pb*S20_318_ and *Pb*TRAP_130_-specific CD8^+^ T cell responses. B6 mice were immunised twice with *Pb* γ-Spz (see Figure 2A). On days 7, 14 and 180 after the last immunisation, *Pb*S20_318_ and *Pb*TRAP_130_-specific CD8^+^ T cell responses were quantified in the spleens and the livers by peptide stimulation followed by ICS. Figure shows flow cytometry plots of IFN-γ co-staining with markers of effector and effector memory phenotypes (CD62L^lo^, CD11a^hi^, CD44^hi^ and CD49d^hi^).

*Pb*S20_318_ and *Pb*TRAP_130_ represent the first reported endogenously processed CD8^+^ T cell epitopes of malaria liver stages in the B6 model. To determine expansion and contraction of *Pb*S20_318_- and *Pb*TRAP_130_-specific CD8^+^ T cells over time, we quantified the responses in the spleen and the liver after one or two immunisations with *Pb* γ-Spz (**Figure 2A**). After a single immunisation, CD8^+^ T cell responses in both the spleen and the liver reach the highest magnitude on day 7 (**Figure 2A,B**). The responses were slightly decreased on day 14 as contraction of the response occurs but they remained quantifiable for up to 180 days after immunisation. The percentages of antigen-specific CD8^+^ T cells were generally higher in the liver that in the spleen. More robust responses were observed after two immunisations with *Pb* γ-Spz (**Figure 2A,B**) Polyfunctional analysis of *Pb*S20_318_- and *Pb*TRAP_130_-specific CD8^+^ T cells revealed the induction of IFN-γ positive cells and IFN-γ/tumour necrosis factor (TNF) double positive CD8^+^ T cells (**Figure S2**). Consistent with the generation of effector and effector memory responses, *Pb*S20_318_ and *Pb*TRAP_130_-specific IFN-γ-producing CD8^+^ T cells were immunophenotyped as CD62L^lo^, CD44^hi^, CD11a^hi^, and CD49d^hi^ (**Figure 3** **, S3**). Cells stimulated with no peptide or cells from naïve mice stimulated with either peptide did not respond to either *Pb*S20_318_ or *Pb*TRAP_130_ (data not shown). Together, these results indicate that immunisation with *Pb* γ-Spz recruits antigen-specific CD8^+^ T cells to undergo differentiation, proliferation, and long-term persistence.

### *Pb*TRAP_130_-specific CD8^+^ T cell responses exhibit *in vivo* cytotoxicity

**Figure 4:**
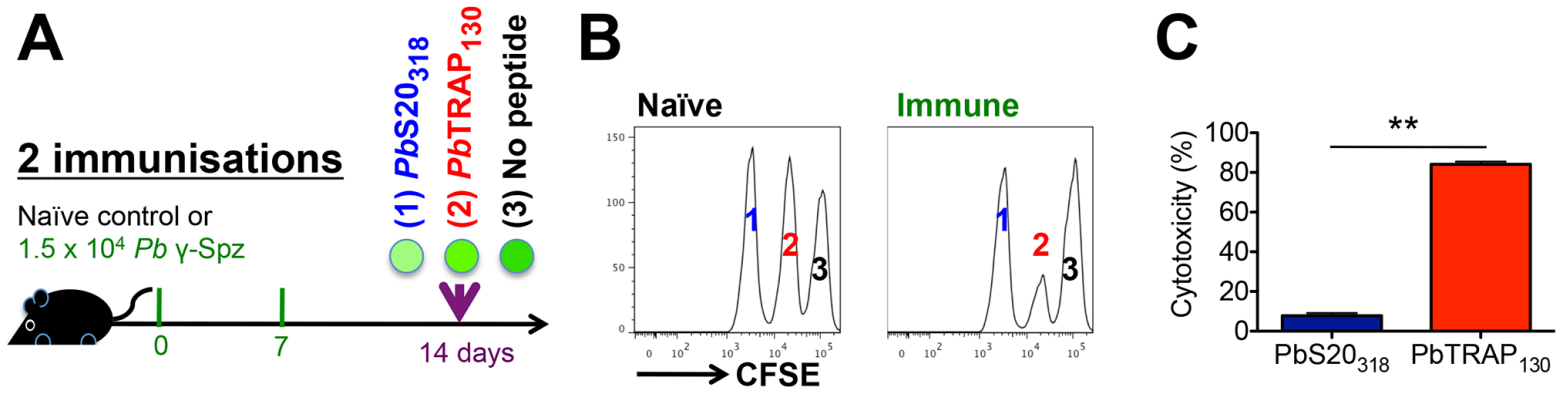
*Pb*TRAP_130_-specific CD8^+^ T cell responses are cytolytic *in vivo*. (**A**) Schematic diagram of methodology. Target cells were prepared by pulsing syngeneic spleen cells with *Pb*S20_318_, *Pb*TRAP_130_, or no peptides prior to labelling with CFSE. Target cells were transferred into naïve or immunised mice 14 days from last *Pb* γ-Spz immunisation. Spleens of recipient mice were harvested 24 hours later and analysed for CFSE fluorescence. (**B**) Representative histogram plots showing the fates of transferred cells in naïve (left) or immune (right) mice. The disappearance of a fluorescent peak signifies cytolysis of labelled splenocytes. (**C**) Quantification of *in vivo* cytolytic activity (**p<0.01, Mann-Whitney test). Figures are representative data from one of 3 experiments with 4 mice/group/experiment.

To determine the *in vivo* cytotoxic potential of *Pb*S20_318_- and *Pb*TRAP_130_-specific CD8^+^ T cells, we utilised an assay that allows the quantification of rapid killing of adoptively transferred target cells by activated CD8^+^ T cells *in vivo* [47]. CFSE-labelled and peptide-pulsed syngeneic targets were transferred to *Pb* γ-Spz-immunised mice 14 days after the last immunisation (**Figure 4A**). We observed considerable (∼90%) disappearance of *Pb*TRAP_130_,-pulsed (**Figure 4B,C**), but not *Pb*S20_318_-pulsed, target cells when transferred to mice that were immunised twice with *Pb* γ-Spz. To corroborate our finding that *Pb*TRAP_130_-specific CD8^+^ T cells exhibit significant cytotoxic activity, we repeated the cell transfer to mice that were immunised only once and to naïve controls (**Figure S4**). Cytotoxicity against cells presenting *Pb*TRAP_130_, but not *Pb*S20_318_, was already apparent after a single immunisation.

### *Pb*TRAP_130_-specific CD8^+^ T cell responses contribute to protection against malaria liver stages

**Figure 5:**
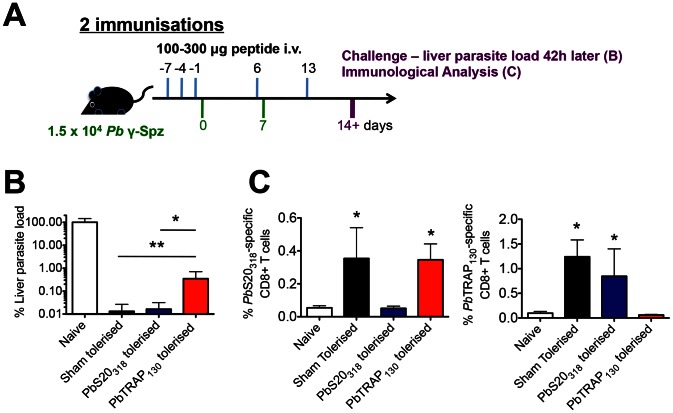
*Pb*TRAP_130_-specific CD8^+^ T cell responses contribute significantly to protection against malaria liver stages. (**A**) Schematic diagram of methodology. Mice were injected with *Pb*S20_318_ or *Pb*TRAP_130_ peptides before and after immunisation with *Pb* γ-Spz. Two weeks after the last immunisation, mice were challenged with sporozoites and the parasite load in the liver was measured 42 hours later. (**B**) Quantification of parasite load in the livers of mice after challenge with sporozoites. Data shown are from two experiments (mean ^+^ SD), *p>0.05 and **p>0.01 (Kruskal-Wallis test/Post-Dunn's test for multiple comparison). (**C**) Spleens of peptide-treated mice were assayed for the presence of *Pb*S20_318_ or *Pb*TRAP_130_-specific CD8^+^ T cells by ICS (*p>0.05, Kruskal-Wallis test/Post-Dunn's test for multiple comparison).

To test whether the newly identified targets of CD8^+^ T cells contribute to protection, we first performed peptide-tolerisation experiments. This method of depleting antigen-specific CD8^+^T cells, being performed in a malaria model for the first time, was adapted from previous studies aimed at inducing and maintaining antigen-specific tolerance [48]–[51]. Mice were subjected to repeated high dose administrations of adjuvant-free *Pb*S20_318_ and *Pb*TRAP_130_ peptides prior to and during the immunisation protocol (two immunisations with *Pb* γ-Spz) (**Figure 5A**). These mice, along with sham-tolerised mice, were challenged with sporozoites and liver parasite loads were measured 42 hours later. As shown in **Figure 5B**, the levels of protection, *i.e.* very low parasite load, in *Pb*S20_318_-tolerised mice were similar to sham-tolerised controls. In contrast, a significantly increased liver parasite load was observed in *Pb*TRAP_130_-tolerised mice. These data indicate that a substantial degree of protection in whole sporozoite-immunised animals, measured by a reduction of parasite liver load over four orders of magnitude, can be attributed to *Pb*TRAP_130_-specific responses.

These results are in agreement with the *in vivo* cytotoxicity experiments (**Figures 4** **, S4**), where we observed the potent *in vivo* cytotoxic function of *Pb*TRAP_130_-specific, but not *Pb*S20_318-_specific, CD8^+^ T cells. However, it remains to be determined whether parasite killing *per se* requires the lytic capacity of antigen-specific CD8^+^ T cells. To corroborate our finding that *Pb*TRAP_130_-specific CD8^+^ T cells contribute to protection against malaria liver stages, the tolerisation experiments were also performed in mice that were immunised only once (**Figure S5A**). As shown in **Figure S5B**, the contribution of *Pb*TRAP_130_,-specific CD8^+^ T cells to protection was already apparent after a single immunisation. Together, our results show a critical and immunodominant contribution of *Pb*TRAP_130_-specific CD8^+^ T cells in parasite killing.

To determine the efficiency of tolerisation, we measured *Pb*S20_318_- and *Pb*TRAP_130_-specific CD8^+^ T cell responses in the tolerised mice (**Figure 5C** **and S5C**). Indeed, in spleens of mice injected with the respective peptides, IFN-γ secretion was greatly reduced and was comparable to naïve mice. Importantly, *Pb*S20_318_-tolerised mice mounted *Pb*TRAP_130_-specific CD8^+^ T cell responses comparable to non-tolerised immune mice. Similarly, *Pb*TRAP_130_-tolerised mice mounted *Pb*S20_318_-specific CD8^+^ T cell responses indistinguishable from controls. These results demonstrate that induction of tolerance was peptide-specific and did not interfere with induction of CD8^+^ T cell responses against other antigens. Of note, the absence of a CD8^+^ T cell response to one antigen did not increase the response to another, suggesting the lack of compensation in the immunodominance hierarchy in our infection model, in contrast to other infections [51]–[54].

At least three immunisations with *Pb* γ-Spz are needed to induce sterile protection in the B6 model [8], [55]. To ascertain whether the development of sterile immunity is dependent on responses to the identified antigens, peptide tolerisation experiments were repeated to mice that were immunised three times with *Pb* γ-Spz (**Table S3**); 14 days after the last immunisation, mice were challenged with sporozoites. Similar to control sham-tolerised mice, multiply immunised *Pb*S20_318_- and *Pb*TRAP_130_-tolerised mice were completely protected and did not develop patent parasitaemia. The results are reminiscent of observations in transgenic Balb/c mice tolerant to T cell responses to *Py*CSP where complete protection was achieved following three immunisations with *Py* γ-Spz despite an immunodominant and protective role for *Py*CSP after one and two immunisations [18]. Taken together, it is likely that additional antigens contribute to protection in both the B6 and Balbc models.

### Vaccination with *Pb*TRAP elicits high levels of *Pb*TRAP_130_-specific CD8^+^ T cell responses

Since *Pb*TRAP_130_-specific, but not *Pb*S20_318_-specific, CD8^+^ T cell responses are cytotoxic *in vivo* and contribute to protection after one or two immunisations with *Pb* γ-Spz, we evaluated the immunogenicity and protective efficacy of *Pb*TRAP in a heterologous prime-boost vaccine regimen with viral vectors. Priming with adenovirus (Ad) carrying a foreign antigen and boosting with orthopoxvirus modified vaccinia Ankara (M) expressing the same antigen has consistently been shown to induce strong T cell responses capable of inducing high levels of efficacy against intracellular pathogens [56]–[58].

**Figure 6:**
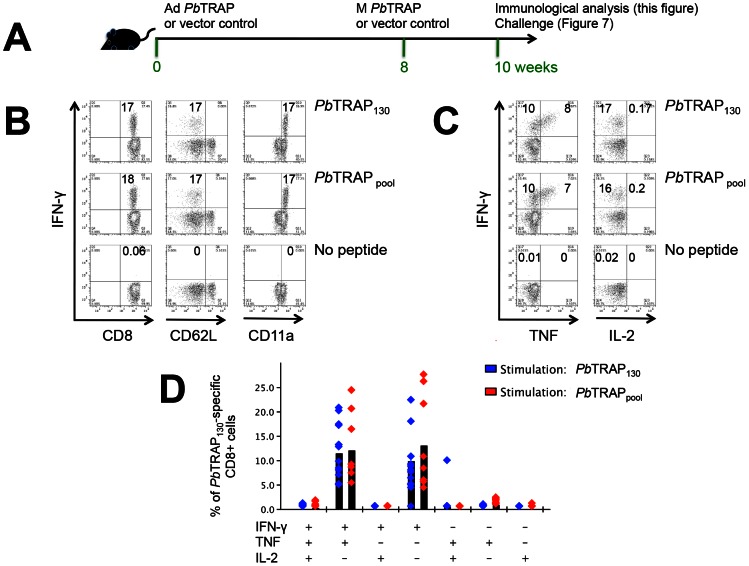
Vaccination with *Pb*TRAP elicits high levels of *Pb*TRAP_130_-specific CD8^+^ T cell responses. (**A**) Schematic diagram of methodology. B6 mice were immunised with Ad *Pb*TRAP (or Ad vector control) and boosted eight weeks later with M *Pb*TRAP (or M vector control). Two weeks later, the frequencies and quality of *Pb*TRAP-specific CD8^+^ T cells on peripheral blood leukocytes were quantified by ICS. (**B**) Flow cytometry plots of IFN-γ co-staining with markers of effector and effector memory phenotypes (CD62L^lo^ and CD11a^hi^). Cells were either not stimulated (no peptide) or stimulated with *Pb*TRAP_130_ (5 µg/ml) or with a pool of 20-mer peptides overlapping by 10 amino acids spanning *Pb*TRAP (*Pb*TRAP_pool_: final concentration is 5 µg/mL for each peptide). (**C**) Flow cytometry plots of IFN-γ co-staining with other effector cytokines, TNF and IL-2. Figures are representative data from one of 4 experiments with 5 mice/group/experiment. (**D**) Polyfunctional analysis of cytokine secretion based on (C). Bars represent the mean value of the % of *Pb*TRAP_130_-specific CD8^+^ cells. Individual data are also shown (blue diamond for *Pb*TRAP_130_ and red diamond for *Pb*S20_318_). Figures B, C and D are representative data from one of at least 4 experiments with 4–5 mice/experiment.

Adenovirus chimpanzee serotype 63 (Ad) and Modified Vaccinia Ankara (M) vaccines expressing a mammalian codon-optimised fragment of *Pb*TRAP were generated. Referred to as Ad-M *Pb*TRAP combination vaccine, they were used to vaccinate B6 mice with an 8-week resting period between priming and boosting (**Figure 6A**). Since the recombinant *Pb*TRAP vaccines contained sequences in addition to *Pb*TRAP_130_, we used both *Pb*TRAP_130_ and a pool of overlapping peptides to *Pb*TRAP (*Pb*TRAP_pool_) in stimulation assays to verify if other *Pb*TRAP-derived sequences were able to induce T cell responses. As shown in **Figure 6B–D**, the frequencies of IFN-γ secreting CD8^+^ T cells were indistinguishable between the two stimulations; approximately ∼17% (range: 14%–50%) of the total CD8^+^ T cells produce IFN-γ specific for *Pb*TRAP_130_ or *Pb*TRAP_pool_. Consistent with the induction of effector responses, these activated IFN-γ-producing cells coincided with the modulation of the corresponding expression markers CD62L^lo^ and CD11a^hi^ (**Figure 6B**). Polyfunctional analysis revealed that the responses were predominantly IFN-γ positive cells and IFN-γ/TNF double positive cells (**Figures 6C,D**). This intracellular cytokine pattern of *Pb*TRAP_130_-specific CD8^+^ T cells were similar to that measured by immunizations with irradiated sporozoites (**Figure S2**). Cells stimulated with no peptide did not respond to either *Pb*TRAP_130_ or *Pb*TRAP_pool_. No cytokine-producing CD4^+^ T cells were detected following *Pb*TRAP_130_ or *Pb*TRAP_pool_ stimulation. These results demonstrated the induction *Pb*TRAP_130_-specific CD8^+^ T cells by vaccination and confirmed that *Pb*TRAP_130_ is the only T cell epitope in *Pb*TRAP in this infection model.

### Protection against sporozoite challenge in Ad-M *Pb*TRAP-immunised mice

**Figure 7:**
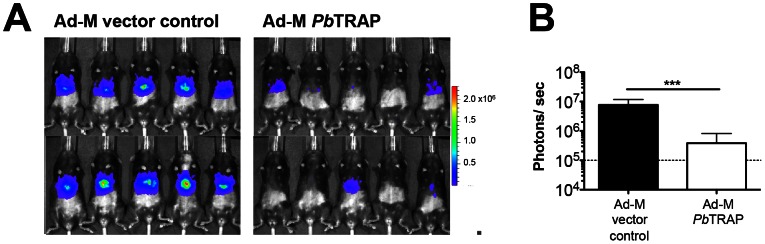
Protective efficacy against sporozoite challenge in Ad-M *Pb*TRAP-immunized mice. Mice immunised with Ad-M *Pb*TRAP (or Ad-M vector control) were challenged with 5×10^3^ *Pb*GFP-Luc_con_ sporozoites (**A**) Imaging of mice 40 hours after challenge and after subcutaneous injection with D-luciferin. (**B**) Quantification of parasite development (***p<0.001, Mann-Whitney test). Dotted lines represent baseline measurement of livers from naïve mice. Figures are representative data from one of 4 experiments with 5 mice/group/experiment.

To determine protective efficacy, mice vaccinated with Ad-M *Pb*TRAP were challenged with *Pb*GFP-Luc_con_. This permitted us to perform *in vivo* imaging in order to quantify hepatic parasite development after challenge and to subsequently follow the development of patent parasitaemia in the same animal. Mice vaccinated with Ad-M *Pb*TRAP show significant efficacy against sporozoite challenge as shown by a considerable decrease (∼95% reduction in liver parasite load; range: 91%–99%) in liver parasite load as compared to mice given Ad-M vector controls (**Figures 7**). However, Ad-M *Pb*TRAP-vaccinated mice did not develop sterile immunity; rather, they developed patent parasitaemia on day 3 after challenge. To further assess vaccine efficacy, we performed survival analysis by measuring time to reach 1% parasitaemia. This measurement has been reported to reflect the number of merozoites that egress from the liver under the assumption that the vaccine has no efficacy against malaria blood stage parasites [44]. Consistent with lower liver parasite load, Ad-M *Pb*TRAP-vaccinated mice showed significant delay in parasite growth as compared to controls (**Figure S6**).

**Figure 8:**
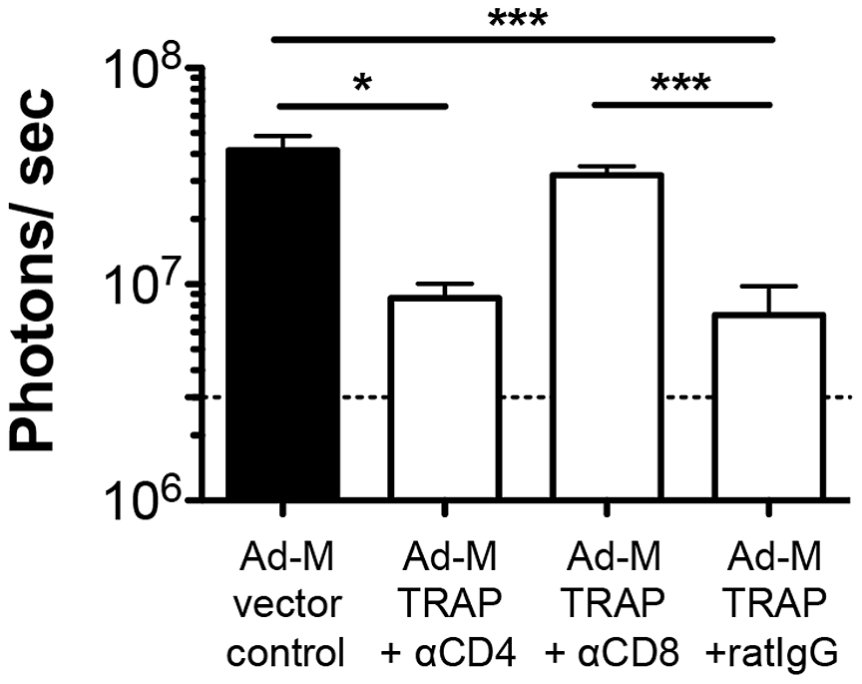
Ad-M *Pb*TRAP immunisation induces CD8^+^ T cell-mediated protective efficacy against sporozoite challenge. Ad-M *Pb*TRAP-immunised mice (n = 7–8 animals/group) were given anti-CD4, anti-CD8 or control (rat IgG) antibodies prior to challenge with 5×10^3^ *Pb*GFP-Luc_con_ sporozoites. The development of parasites in the liver was quantified by live luminescence (*p<0.05, **p<0.01, ***p<0.001, Kruskal-Wallis test/Post-Dunn's test for multiple comparison). Dotted lines represent baseline measurement of livers from naïve mice.

Finally, to establish whether the observed decrease in liver parasite load in mice vaccinated with Ad-M *Pb*TRAP is solely mediated by CD8^+^ T cells, groups of vaccinated mice were administered CD8^+^ depleting antibodies or control rat IgG prior to sporozoite challenge (**Figure 8**). In addition, a group of mice vaccinated with Ad-M *Pb*TRAP were given anti-CD4^+^ T cell antibodies. Efficacy was abrogated in the group that received anti-CD8^+^ T cell, but not anti-CD4 T cell, antibodies after vaccination. Taken together, these results provide a striking correlation between the high levels of *Pb*TRAP_130_-specific CD8^+^ T cells and the CD8^+^ T cell-mediated efficacy elicited by Ad-M *Pb*TRAP vaccination.

## Discussion

### Vaccination with malaria parasites elicits few protective T cell epitopes

Over 40 years have passed since the observation that immunisation of mice with a whole organism vaccine, i.e. *Pb* γ-Spz, induces complete protection against normal sporozoite challenge [3]. Although a crucial role for CD8^+^ T cells in this protection was demonstrated more than 25 years ago [10], [11], information regarding the precise targets of these cells remains remarkably incomplete. This paucity of naturally processed parasite-specific epitopes of CD8^+^ T cell responses has thwarted efforts to characterise the induction of protective and possibly non-protective responses that can be rigorously studied in murine vaccination models for malaria liver stages [59]. CSP, the major protein that coats the sporozoite's surface, has been at the forefront of malaria vaccination studies for more than two decades, and CSP-specific responses have been the benchmark in measuring cellular responses to malaria liver stages [20]. However, *Pb*CSP and *Py*CSP are targets of immunodominant and protective CD8^+^ T cell responses only in the BALB/c model [16], [60]. The need to identify non-CSP targets of CD8^+^ T cell responses comes from accumulating evidence suggesting that protection against malaria infection can be obtained in the absence of T cell responses to CSP [18], [19].

In this study, we employed an unbiased approach for screening *Pb*-derived peptides that are recognised by CD8^+^ T cells from B6 mice immunised with whole sporozoite strategies, which are known to induce protection. Out of 600 peptides predicted to contain H-2^b^ motifs, we report for the first time the identification of *two* peptides as signature targets of CD8^+^ T cells from sporozoite-immunised B6 mice. Notably, we provide evidence that responses to *Pb*TRAP confers partial efficacy *in vivo*. Thus, we anticipate that pre-clinical development of an anti-malarial vaccine based on whole sporozoites or sub-unit vaccines that incorporate TRAP will be facilitated by the identification of quantifiable signatures of effective immunisation.

*Plasmodium* parasites are complex pathogens with a ∼23 Mb genome [32], [61]. The identification of targets of CD8^+^ T cells has important implications for understanding the hierarchy and the scope of responses to complex pathogens. When we initiated this study, we expected the CD8^+^ T cell responses to be relatively widely dispersed over many sporozoite and liver stage antigens that together can account for the protection offered by sporozoite immunisation. However, we only identified two targets - *Pb*S20_318_ and *Pb*TRAP_130_. Our inability to identify additional targets is indicative of immune evasion once sporozoites invade target cells, a hallmark that might generally apply to parasitic infections. Coincidentally, vaccines against these complex pathogens remain elusive. In marked contrast, a similar epitope prediction approach revealed broad and abundant H2^b^-restricted CD8^+^ T cell epitopes of vaccinia virus [22].

One potential limitation in our *in silico* analysis is the inclusion of only the top hits of potential liver stage peptides. In addition, by utilising IFN-γ as a read-out of our assays, we cannot formally exclude other cytokines or cellular markers that may serve as additional signatures of protection. The quantification of IFN-γ expression by CD8^+^ T cells is a reliable read-out in murine and human vaccine studies. Consistent with the observation that few antigens are recognised by CD8^+^ T cells from immunised mice, an assessment of 34 candidate non-CSP sporozoite antigens in the BALB/c model failed to reveal any additional epitopes [62]. In experimental studies in humans, antigenic analysis of genomic and proteomic data revealed 16 new antigens recognised by volunteers immunised with *Pf* γ-Spz [33]. However, there was no significant overall difference between protected and non-protected volunteers, indicating that T cell proliferation is associated with pathogen exposure rather than protection.

### Immunodominant epitopes diverge fundamentally in cytolytic efficacy

By measuring the responses to *Pb*S20_318_ and *Pb*TRAP_130_, we report the first characterisation of the development of CD8^+^ T cell responses following sporozoite immunisation in B6 mice. *Pb*S20_318_- and *Pb*TRAP_130_-specific CD8^+^ T cells persist to long-term memory while displaying effector and effector memory phenotypes in both the spleens and livers of immunised mice. The ability of *Pb*S20_318_ and *Pb*TRAP_130_-specific CD8^+^ T cells to produce IFN-γ after peptide stimulation, coinciding with activation phenotypes, suggested their ability to exert cytotoxic functions *in vivo*. Remarkably, only *Pb*TRAP_130_-specific, but not *Pb*S20_318_-specific, CD8^+^ T cells, are able to lyse target cells pulsed with the respective peptides. These results provide the first evidence that liver stage infection evokes antigen-specific CD8^+^ T cells that are both cytolytic and non-cytolytic. In support of this notion, tolerisation induction via high dose intravenous injection of peptides revealed that *Pb*TRAP_130_-specific, but not *Pb*S20_318_-specific, CD8^+^ T cells contribute to parasite killing.

Owing to the complexity of immune responses to sporozoites, it was not surprising that *Pb*TRAP_130_-tolerised mice that received multiple immunisations with *Pb* γ-Spz were completely protected against sporozoite challenge. The residual efficacy obtained in the challenged *Pb*TRAP_130_-tolerised mice is likely due to responses to unidentified targets of both CD8^+^ and CD4^+^ T cells. It is noteworthy that in the *Py*-B6 and *Py*-C57Bl/10 (both expressing H2^b^) models, both CD8^+^ and CD4^+^ T cells equally participate in the inhibition of parasite development [63]. The results are in agreement with earlier findings on the immunodominant but imperfect CSP epitope in the Balb/c infection model [18].

In our prime-boost vaccination experiments with Ad-M *Pb*TRAP in B6 mice, we were able to generate very high levels (14–50%) of circulating *Pb*TRAP_130_-specific CD8^+^ T cells, yet complete protection was not achieved. This level of antigen-specific CD8^+^ T cells is not achieved following multiple immunisations with *Pb* γ-Spz in B6 mice, yet complete protection is attained (Figure 2). It is likely that additional antigens help consolidate the protection afforded by whole sporozoite immunisation. Very high levels of *Py*CSP- or *Pb*CSP-specific CD8^+^ T cells generated by various vaccination strategies in Balb/c mice have been shown to induce complete protection [64]–[69]. We anticipate that generating high levels of *Pb*S20_318_-specific CD8^+^ T cells through a similar Ad-M vaccine protocol is unlikely to confer any quantifiable protection in B6 mice. Further studies are needed to characterise the complex mechanisms of protection. In a recent study, it was suggested that strain-specific background genes in nonhematopoietic cells can control the threshold of antigen-specific CD8 T cells necessary for protection [70].

Taken together, our results raise the intriguing and important question as to what factors govern the protective efficacy of responding antigen-specific CD8^+^ T cells. Numerous possibilities exist to explain these findings, including quantitative and functional differences in CD8^+^ T cells, distinct expression of cognate proteins and/or MHC class I presentation. Future work is warranted to identify the underlying mechanisms that distinguish cellular correlates of sporozoite exposure (*Pb*S20_318_-specific CD8^+^ T cells), from signatures of protection (*Pb*TRAP_130_-specific CD8^+^ T cells). Our work suggests that the mechanisms involved can now be studied in a tractable animal model.

### Implications for malaria subunit vaccine development

The outcome of our work has obvious relevance for vaccine development. We provide a clearly defined model system, in which to investigate fundamental aspects of the CD8^+^ T cell response and to manipulate this response to enhance protective immunity. Our identification of TRAP as major target of protective CD8^+^ T cells in the *Pb*-B6 model lends strong support for *Pf*TRAP as leading vaccine candidate to elicit strong cellular immune responses. Initial and partial results from ongoing phase III clinical trials of the RTS,S/AS01 malaria vaccine candidate, which is based on *Pf*CSP, demonstrated only modest efficacy [71], [72]. RTS,S/AS01 immunisation elicits high concentrations of anti-CSP antibodies [73] and induces CD4^+^, but not CD8^+^, T cell responses [74]. Towards a second-generation, >80% effective malaria vaccine, rational vaccine design to elicit superior and lasting immune responses is critical.

TRAP has been identified as a viable target for *Pf* vaccines. While *Py*TRAP was suggested as a target of CD8^+^ T cells in BALB/c mice [66], [75], the epitopes have been elusive so far. *Pf*TRAP induces large numbers of polyfunctional CD8^+^ T cells in experimental vaccination studies [76], and these T cells are associated with partial but significant efficacy in human vaccine trials [43], [45]. However, when tested in larger Phase IIb trials in endemic areas, efficacy was lost despite moderate immunogenicity of the vaccine, suggesting that the T cell response that is induced is insufficient in some way [46]. Further work is needed to improve the immunogenicity of TRAP-based vaccines for malaria-exposed individuals. More recently, vaccination regimes with viral vectors have induced much stronger CD8^+^ T cell responses in phase I trials [76] and greater efficacy in controlled challenge studies (Ewer et al., submitted for publication). Based on our identification of a major protective CD8^+^ T cell epitopes in *Pb*TRAP, we propose that the parasite-host combination *Pb*-B6 is an attractive and relevant model to further systematically explore subunit vaccination strategies, focusing on the TRAP antigen with the aim of increasing potent and durable sterilising immunity.

## Materials and Methods

### Ethics and animal experimentation

Animal procedures were performed in accordance with the German ‘Tierschutzgesetz in der Fassung vom 18. Mai 2006 (BGBl. I S. 1207)’, which implements the directive 86/609/EEC from the European Union and the European Convention for the protection of vertebrate animals used for experimental and other scientific purposes. Animal protocols were approved by the ethics committee and the Berlin state authorities (LAGeSo Reg# G0469/09). Experiments performed at the University of Oxford were performed under license from the United Kingdom Home Office under the Animals (Scientific Procedures) Act 1986 and approved by the Animal Care and Ethical Review Committee. Female B6 mice 6–8 weeks of age were purchased from either Charles River (Sulzfeld, Germany) or Harlan (Derbyshire, UK).

### Pb ANKA inoculations

The complete life cycles of two parasite strains, *Pb* (strain ANKA, clone cl15cy1) and *Pb*GFP-Luc_con_ (strain ANKA, clone 676m1cl1), were previously cloned and maintained by continuous cycling between rodent hosts and *Anopheles stephensi* mosquitoes [77]. Sporozoites were isolated from salivary glands. For *Pb* γ-Spz, the radiation dose was 1.2×10^4^ cGy, and mice were immunised intravenously with 1.5×10^4^ parasites. For *Pb* infection and treatment strategies, 1.5×10^4^ sporozoites were injected intravenously while anti-malaria drugs were given intraperitoneally: 160 mg AZ/kg (days 0, 1 and 2) [8], 60 mg PQ/kg (days 0, 1 and 2) [7] and 40 mg CQ/kg (days 0–7) [6]. For *Pb*HKSpz, parasites were subjected to 95°C for 15 minutes [38]. Immunised mice were challenged with 10^4^ *Pb* sporozoites. In some experiments involving imaging, mice were challenged with 5×10^3^ *Pb*GFP-Luc_con_ normal sporozoites. For mice receiving >1 immunisations, sporozoites were administered >7 days apart.

### Peptides and peptide treatments

Peptide libraries were generated by solid phase synthesis (Peptides and Elephants, Potsdam). *Pb*S20_318_ (VNYSFLYLF) and *Pb*TRAP_130_ (SALLNVDNL) peptides were additionally synthesised at large scale. Peptides were synthesised as peptide amides and lyophilised peptides were resuspended in DMSO at a concentration of at least 1 mg/ml and stored at −80°C. Purity was confirmed by mass spectrometry. Peptides were diluted in PBS and tested individually at 10 µg/ml for the ELISpot assay and T cell stimulations with the exception of Figure 6 in which peptides were used at 5 µg/ml. A 20-mer peptide library (pooled) spanning the entire sequence of *Pb*TRAP was generated, in which each peptide was overlapped 10 amino acids to another peptide (Mimotopes, Victoria, Australia). The final concentration of the peptide pool used for stimulations was 5 µg/ml for each peptide. For the tolerisation experiments, mice received 300 µg peptide (either *Pb*S20_318_ or *Pb*TRAP_130_) on day −7 and 100 mg on days −4 and −1 as described [51]. Mice were immunised on day 0 and received 100 µg peptide weekly until the end of the experiment.

### Recombinant vectored vaccines

AdCh63 and MVA vaccines expressing a mammalian codon-optimised fragment of *Pb*TRAP [39] (GeneArt, Regensburg, Germany) were constructed and propagated based on previously published viral vectors [76], [78]. The TRAP signal peptide sequence was replaced with the human tissue plasminogen activator signal peptide sequence and the 3′ transmembrane region deleted. The viral vectors, referred to as Ad-M *Pb*TRAP as a combination vaccine, were administered intramuscularly in endotoxin-free PBS at a concentration of 10^9^ viral particles for Ad *Pb*TRAP and 10^6^ plaque-forming units for M *Pb*TRAP.

### Quantification of antigen-specific CD8^+^ T cell responses

ELiSpot assay was performed as described [35] with minor modifications. CD8^+^ T cells were purified from spleen cells by positive selection using mouse CD8 microbeads (MACS Miltenyi Biotec, Bergish Gladbach, Germany). Syngeneic spleen cells from naïve B6 mice were coated with peptides and were used as antigen-presenting cells. Anti-IFN-γ [AN18] and biotin-anti-IFN-γ [R4-6A2] were obtained from Mabtech (Nacka Strand, Sweden).

For T cell stimulations followed by ICS, splenic and liver-infiltrating lymphocytes were incubated with peptides for 5–6 hours in the presence of Brefeldin A (eBioscience, California, USA), followed by standard surface and intracellular staining procedures. Data was acquired using either a LSRII or a LSRFortessa (BD Bioscience, Heidelberg/Oxford). Antibodies for stainings were obtained from eBioscience: anti-mouse CD8 [53-6.7], CD62L [MEL14], CD44 [IM7], CD11a [M17/4], CD49d [R1-2], IFN-γ [R4-6A2], TNF [MP6-XT22] and interleukin (IL)-2 [JES6-5H4].

Data analysis was performed using FlowJo 7.6.3 (Tree Star Inc., Oregon, USA). SPICE 5.21, a gift from Dr. Mario Roederer (NIAID, NIH, Maryland, USA) was used to analyse polyfunctional data.

### In vivo cytotoxicity assay

The cytotoxic potential of antigen-specific CD8^+^ T cells was assayed as described [47].

### Quantification of parasite development after challenge

Livers were excised 42 hours after challenge and total RNA was isolated. cDNAs, generated by reverse transcription, were used as templates for quantitative real-time PCR [8] of *Pb* 18S rRNA (gi: 160641) and GAPDH sequences (gi: 281199965) using the Applied Biosystem Step One Plus Real Time PCR System (Darmstadt, Germany). Relative parasite loads were calculated using the ΔΔ*C_t_* method.

For *in vivo* imaging, an IVIS200 imaging system (Caliper Life Sciences, Chesire, United Kingdom) was utilised to monitor parasite development in the liver 42 hours after challenge [78]. Mice were anaesthetised, injected subcutaneously with D-luciferin (Synchem Laborgemeinschaft OHG, Felsberg/Altenberg) (100 mg/kg in PBS), and 8 minutes later, imaged for 120 s at binning value of 8 and fields-of-view (FOV) of 12.8 cm. Bioluminescence in the liver was quantified using Living Imaging 4.2 software (Caliper Life Science) and expressed as total flux of photons per second of imaging time.

The development of patent parasitaemia was determined based on Giemsa-stained blood smears. Relationships between log percentage parasitemia and time after challenge were plotted. Kaplan Meier analysis was performed to compare the parasite growth rate, and protection was measured as a delay in reaching 1% parasitaemia [44].

### Statistics

Statistical analysis (see Figure Legends), unless otherwise specified, was performed using Prism 5.0c (GraphPad Software Inc., CA, USA). Mixture model calculations were performed using Stata 12 (StataCorp LP, TX, USA).

## Supporting Information

Figure S1 **Initial characterisation of** ***Pb*** **S20_318_ and** ***Pb*** **TRAP_130_.** Schematic diagrams of *Pb*S20 and *Pb*TRAP, and the location of the identified CD8^+^ T cell determinants. Genetically mobile domains and domain architectures were analysed using the Simple Modular Architecture Research Tool (SMART - http://smart.emblheidelberg.de/).
(TIF)

Figure S2 **Polyfunctional analysis of** ***Pb*** **S20_318_ and** ***Pb*** **TRAP_130_-specific CD8^+^ T cells in the spleen after one or two immunisations with** ***Pb*** **γ-Spz.** Data is based on Figure 2. Bars represent the mean value of the % of antigen-specific CD8^+^ cells. Individual data are also shown.
(TIF)

Figure S3 **Phenotyping** ***Pb*** **S20_318_ and** ***Pb*** **TRAP_130_-specific CD8^+^ T cell responses (one immunisation).** B6 mice were immunised once with *Pb* γ-Spz similar to that in Figure 2. On days 7, 14 and 180 after the last immunisation, *Pb*S20_318_ and *Pb*TRAP_130_-specific CD8^+^ T cell responses were quantified in the spleens and the livers by peptide stimulation followed by ICS. Figure shows flow cytometry plots of IFN-γ co-staining with markers of effector and effector memory phenotypes (CD62L^lo^, CD11a^hi^, CD44^hi^ and CD49d^hi^).
(TIF)

Figure S4 ***Pb*** **TRAP_130_-specific CD8^+^ T cell responses are cytolytic** ***in vivo*** **(one immunisation).** (**A**) Schematic diagram of methodology. Target cells were prepared by pulsing syngeneic spleen cells with *Pb*S20_318_, *Pb*TRAP_130_, or no peptides prior to labelling with CFSE. Target cells were transferred into naïve or mice immunised 14 days earlier with *Pb* γ-Spz. Spleens of recipient mice were harvested 24 hours later and analysed for CFSE fluorescence. (**B**) Representative histogram plots showing the fates of transferred cells in naïve (left) or immune (right) mice. The disappearance of a fluorescent peak signifies cytolysis of labelled splenocytes. (**C**) Quantification of *in vivo* cytolytic activity (**p<0.01, Mann-Whitney test). Figures are representative data from one of 3 experiments with 4 mice/group/experiment.
(TIF)

Figure S5 ***Pb*** **TRAP_130_-specific CD8^+^ T cell responses contribute significantly to protection against malaria liver stages (one immunisation).** (**A**) Schematic diagram of methodology. Mice were injected with *Pb*S20_318_ or *Pb*TRAP_130_ peptides before and after immunisation with *Pb* γ-Spz. Two weeks after immunisation, mice were challenged with sporozoites and the parasite load in the liver was measured 42 hours later. (**B**) Quantification of parasite load in the livers of mice after challenge with sporozoites. Data shown are from two experiments (mean ^+^ SD), *p>0.05 and **p>0.01 (Kruskal-Wallis test/Post-Dunn's testfor multiple comparison). (**C**) Spleens of peptide-treated mice were assayed for the presence of *Pb*S20_318_ or *Pb*TRAP_130_-specific CD8^+^ T cells by ICS (*p>0.05, Kruskal-Wallis test/Post-Dunn's test for multiple comparison).
(TIF)

Figure S6 **Protective efficacy against normal sporozoite challenge in Ad-M** ***Pb*** **TRAP-immunized mice: analysis of time to patent parasitaemia.** Figure shows Kaplan-Meier plots comparing time with patent parasitemia (by blood film) in Ad-M *Pb*TRAP and Ad-M vector control-immunised mice. Data is based on Figure 7. Differences between two groups were analysed using the Log-rank (Mantel Cox) test (***p>0.001).
(TIF)

Table S1 **Summary of datasets used in the epitope analysis.**
(PDF)

Table S2 **Complete list of synthesised peptides.**
(PDF)

Table S3 **Tolerisation and multiple immunisation with** ***Pb*** **γ-Spz.**
(DOCX)
