# Supplementary figures and images for: Identification of Targets of CD8+ T Cell Responses to Malaria Liver Stages by Genome-wide Epitope Profiling
Source: PLoS Pathog. 2013 May 9;9(5):e1003303. doi: 10.1371/journal.ppat.1003303 (PMC3649980; doi:10.1371/journal.ppat.1003303)

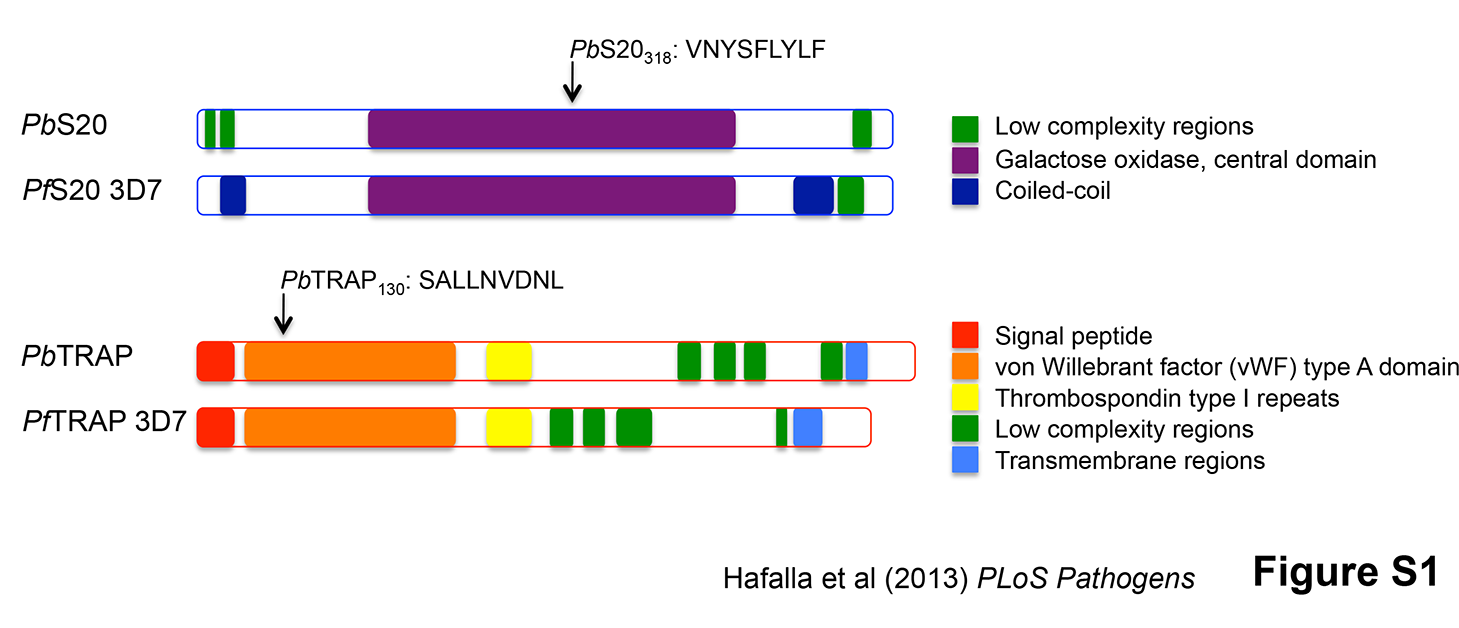

Supplement: Figure S1 — Initial characterisation of Pb S20318 and Pb TRAP130. Schematic diagrams of PbS20 and PbTRAP, and the location of the identified CD8+ T cell determinants. Genetically mobile domains and domain architectures were analysed using the Simple Modular Architecture Research Tool (SMART - http://smart.emblheidelberg.de/). (TIF) [file ppat.1003303.s001.tif]

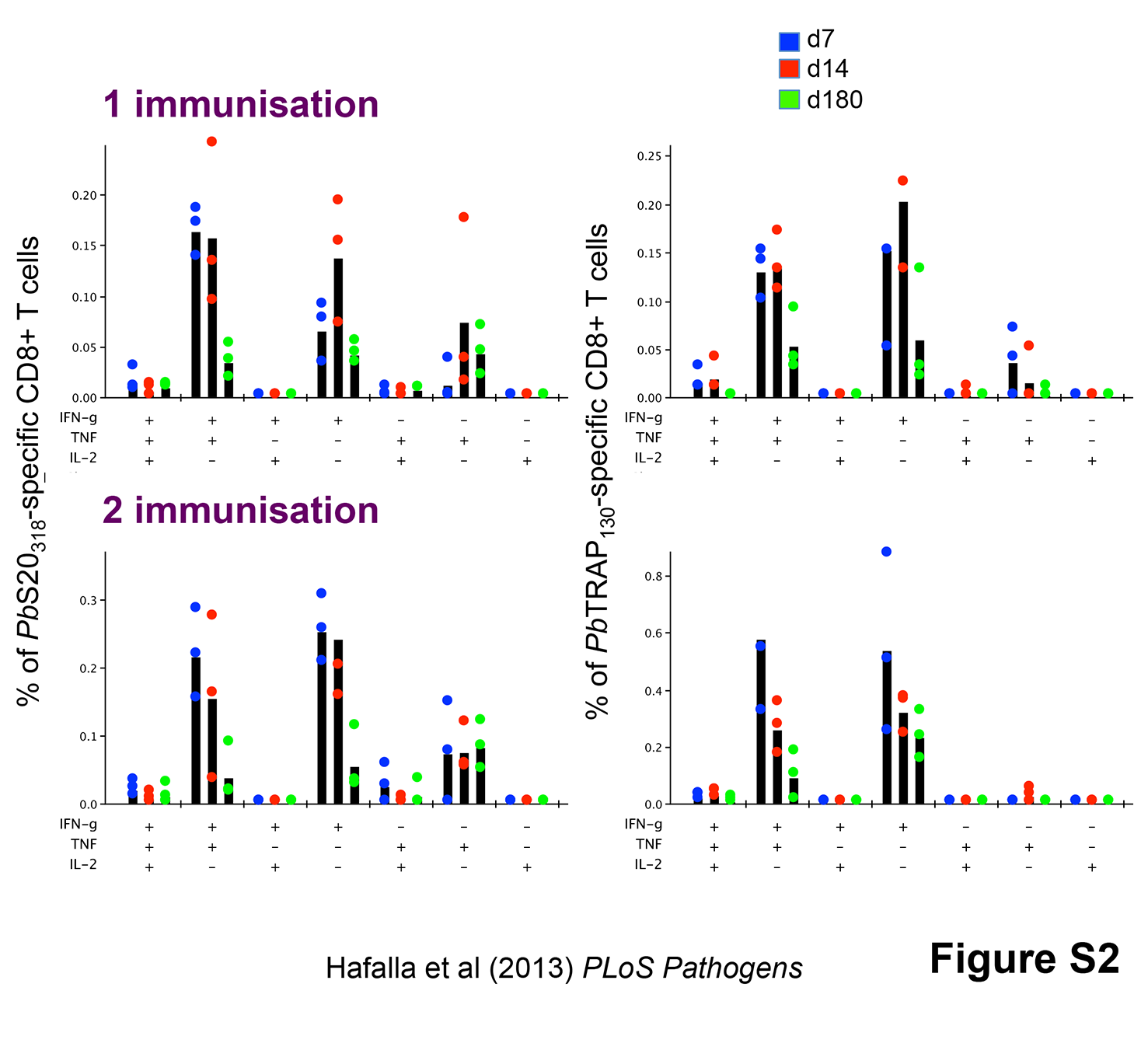

Supplement: Figure S2 — Polyfunctional analysis of Pb S20318 and Pb TRAP130-specific CD8+ T cells in the spleen after one or two immunisations with Pb γ-Spz. Data is based on Figure 2. Bars represent the mean value of the % of antigen-specific CD8+ cells. Individual data are also shown. (TIF) [file ppat.1003303.s002.tif]

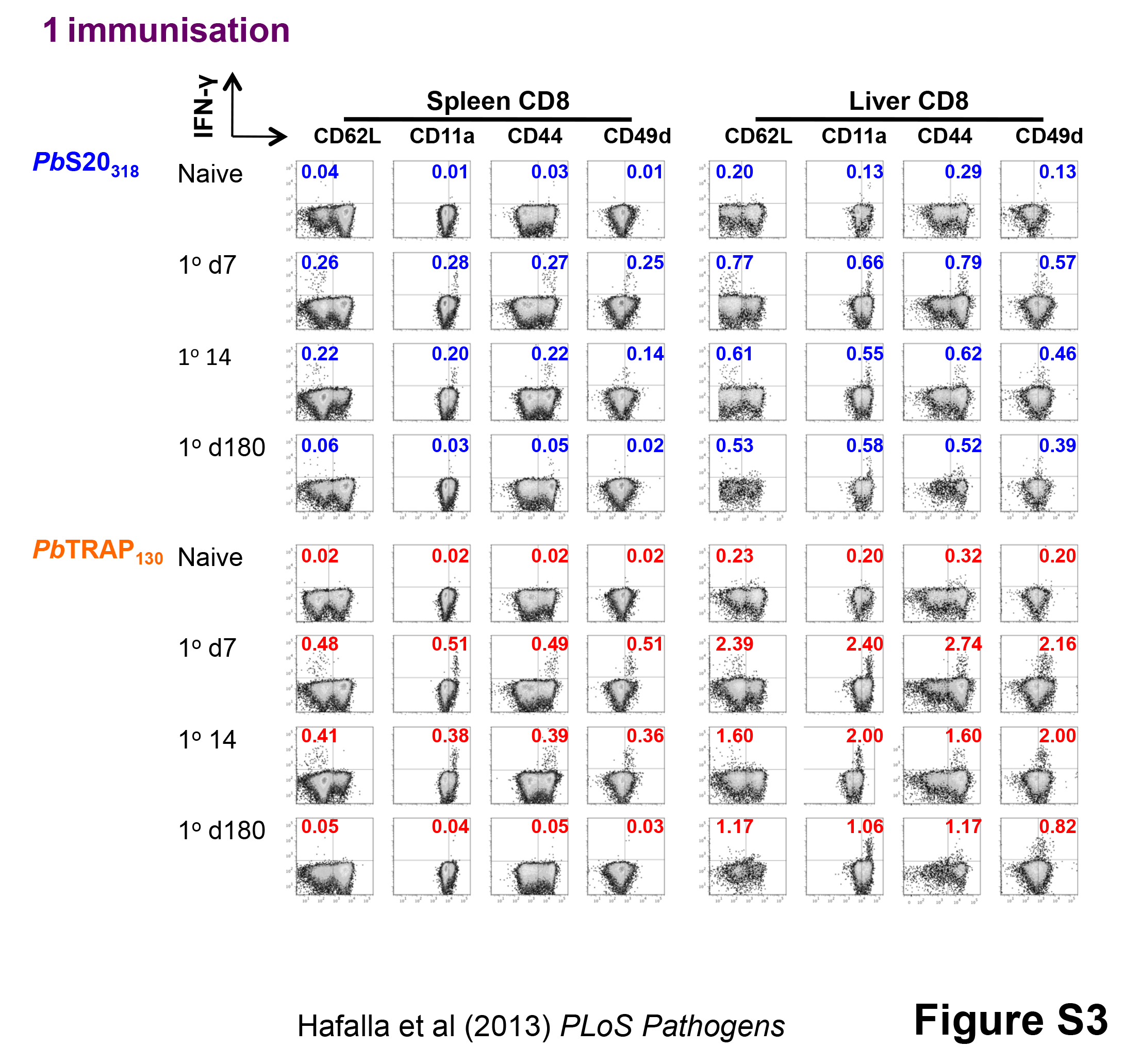

Supplement: Figure S3 — Phenotyping Pb S20318 and Pb TRAP130-specific CD8+ T cell responses (one immunisation). B6 mice were immunised once with Pb γ-Spz similar to that in Figure 2. On days 7, 14 and 180 after the last immunisation, PbS20318 and PbTRAP130-specific CD8+ T cell responses were quantified in the spleens and the livers by peptide stimulation followed by ICS. Figure shows flow cytometry plots of IFN-γ co-staining with markers of effector and effector memory phenotypes (CD62Llo, CD11ahi, CD44hi and CD49dhi). (TIF) [file ppat.1003303.s003.tif]

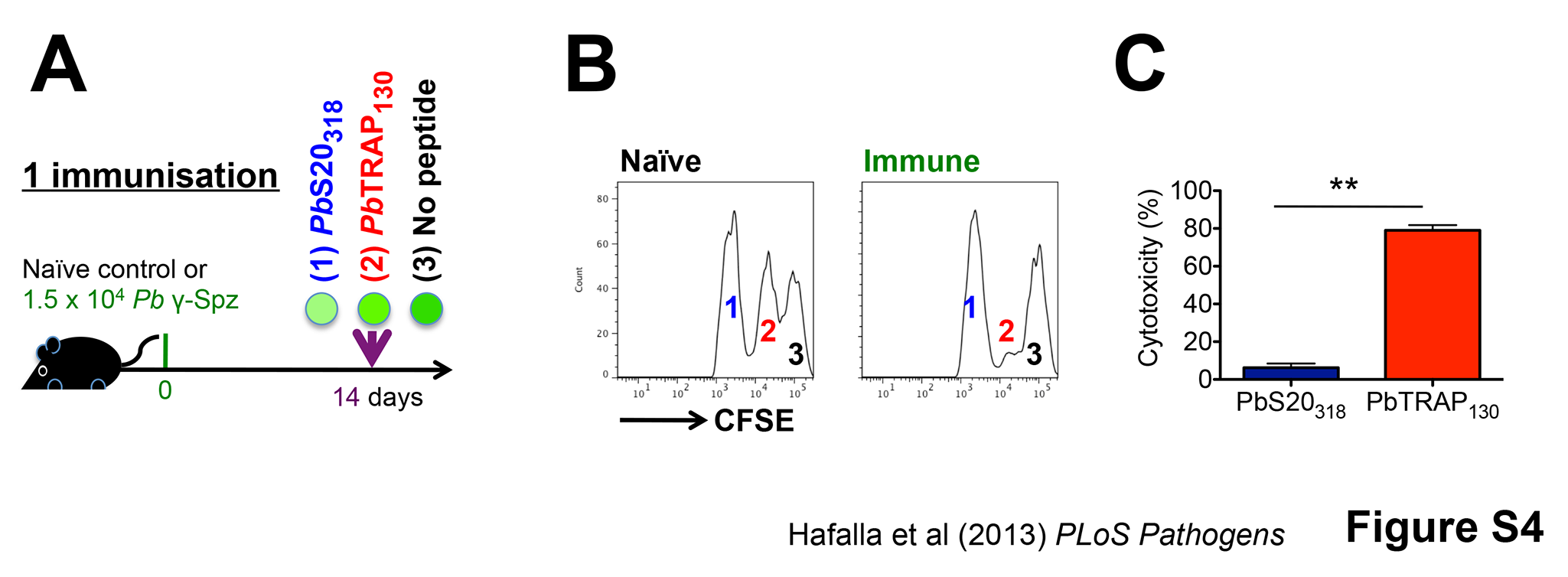

Supplement: Figure S4 — Pb TRAP130-specific CD8+ T cell responses are cytolytic in vivo (one immunisation). (A) Schematic diagram of methodology. Target cells were prepared by pulsing syngeneic spleen cells with PbS20318, PbTRAP130, or no peptides prior to labelling with CFSE. Target cells were transferred into naïve or mice immunised 14 days earlier with Pb γ-Spz. Spleens of recipient mice were harvested 24 hours later and analysed for CFSE fluorescence. (B) Representative histogram plots showing the fates of transferred cells in naïve (left) or immune (right) mice. The disappearance of a fluorescent peak signifies cytolysis of labelled splenocytes. (C) Quantification of in vivo cytolytic activity (**p<0.01, Mann-Whitney test). Figures are representative data from one of 3 experiments with 4 mice/group/experiment. (TIF) [file ppat.1003303.s004.tif]

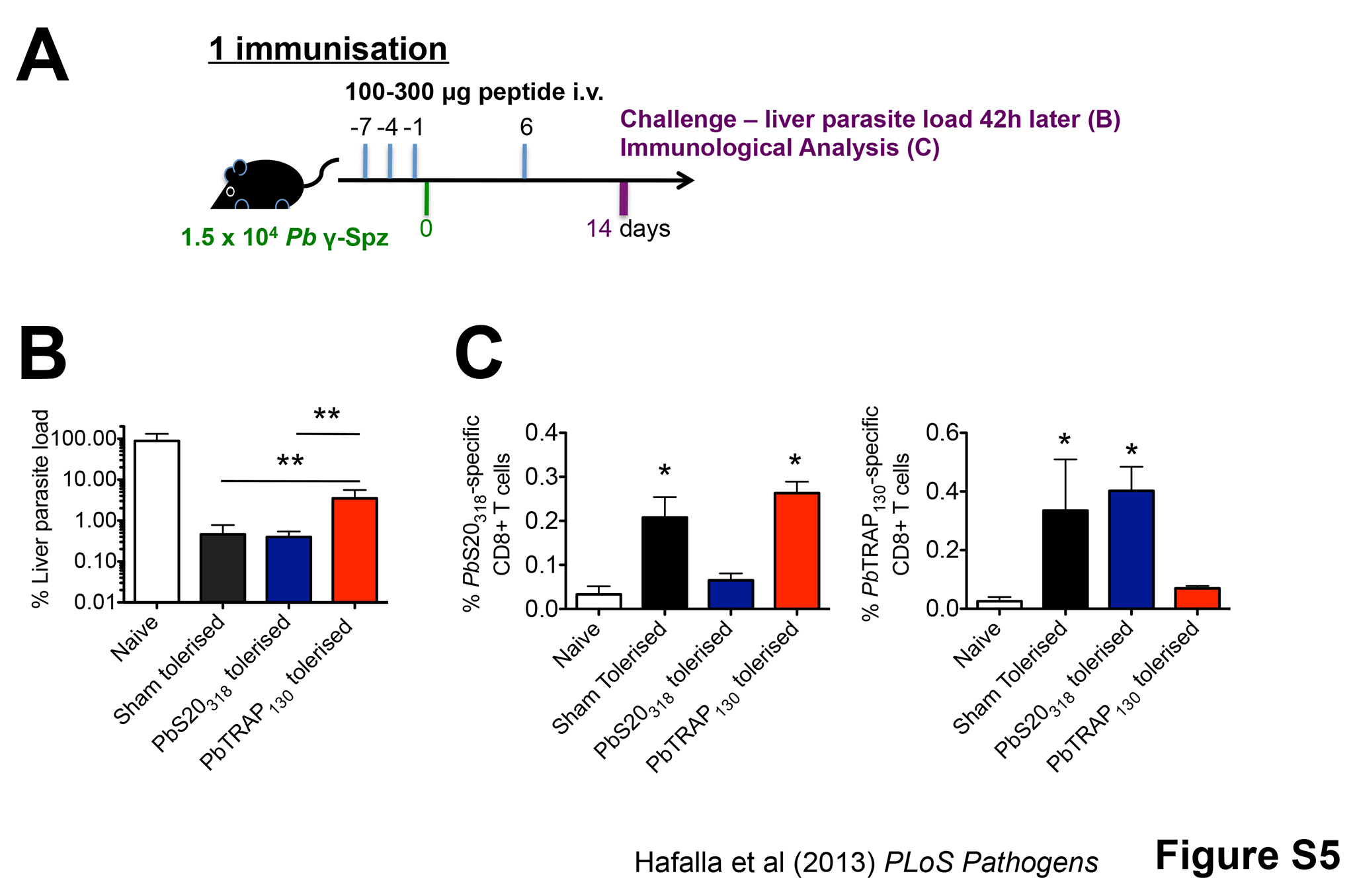

Supplement: Figure S5 — Pb TRAP130-specific CD8+ T cell responses contribute significantly to protection against malaria liver stages (one immunisation). (A) Schematic diagram of methodology. Mice were injected with PbS20318 or PbTRAP130 peptides before and after immunisation with Pb γ-Spz. Two weeks after immunisation, mice were challenged with sporozoites and the parasite load in the liver was measured 42 hours later. (B) Quantification of parasite load in the livers of mice after challenge with sporozoites. Data shown are from two experiments (mean + SD), *p>0.05 and **p>0.01 (Kruskal-Wallis test/Post-Dunn's testfor multiple comparison). (C) Spleens of peptide-treated mice were assayed for the presence of PbS20318 or PbTRAP130-specific CD8+ T cells by ICS (*p>0.05, Kruskal-Wallis test/Post-Dunn's test for multiple comparison). (TIF) [file ppat.1003303.s005.tif]

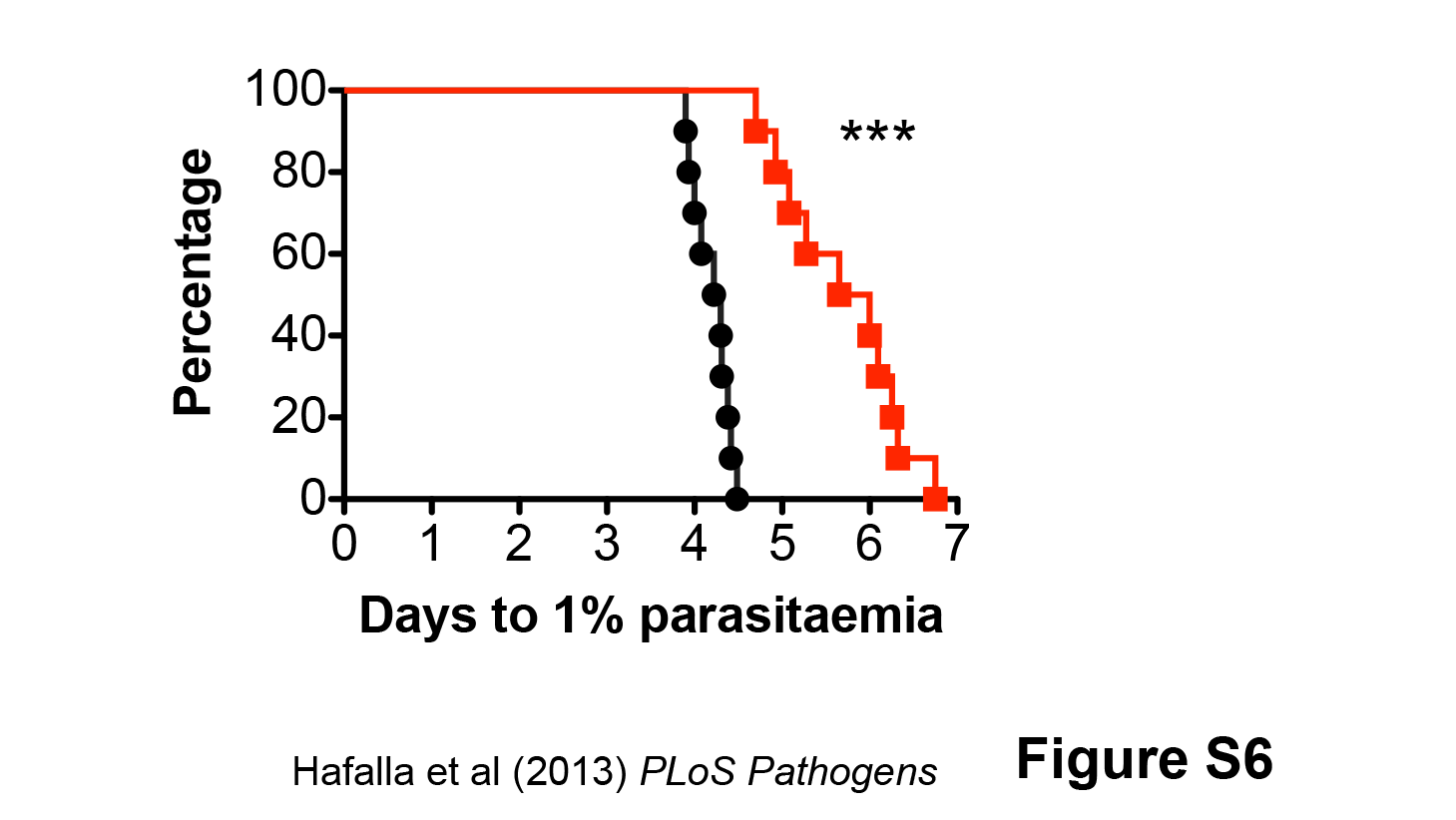

Supplement: Figure S6 — Protective efficacy against normal sporozoite challenge in Ad-M Pb TRAP-immunized mice: analysis of time to patent parasitaemia. Figure shows Kaplan-Meier plots comparing time with patent parasitemia (by blood film) in Ad-M PbTRAP and Ad-M vector control-immunised mice. Data is based on Figure 7. Differences between two groups were analysed using the Log-rank (Mantel Cox) test (***p>0.001). (TIF) [file ppat.1003303.s006.tif]
