# Supplementary material for: Identification of Targets of CD8+ T Cell Responses to Malaria Liver Stages by Genome-wide Epitope Profiling
Source: PLoS Pathog. 2013 May 9;9(5):e1003303. doi: 10.1371/journal.ppat.1003303 (PMC3649980; doi:10.1371/journal.ppat.1003303)
Supplement: Table S1 — Summary of datasets used in the epitope analysis. (PDF) [file ppat.1003303.s007.pdf]

TABLE S1: The different data sets used in this study

	Data sets [1]	References[8]	Proteins covered	H2 alleles analysed	Top unique peptides synthesised[9]
1	'GOP [2]', UIS [3], S [4], Axenic and LSA homologues [5]	Matuschewski et al., 2002b<br>Kaiser et al., 2004<br>Wang et al., 2004	75	K^b and D^b	115
2	SAGE of SG Spz[6]	Rosinski-Chupin et al., 2007	123	K^b and D^b	118
3	Liver Stage Transcriptome	Tarun et al., 2008	534	K^b and D^b	90
4	Whole Genome [7]	Hall et al., 2005	all	K^b and D^b	176
5	Human Correlates 1	Doolan et al., 2003	27	K^b and D^b	32
6	Human Correlates 2 and MSP-1	Doolan et al., 2008	15	K^b and D^b	69

(1) The sequences included in the analyses were those downloaded from PLASMODB from April – December 2010. *Pb* orthologues were used when original data sets were from *Py*.
(2) 'GOP' – **G**rand **O**ld **P**roteins – consisting of previously studied antigens CSP, TRAP, HEP17, AMA-1, SPECT, p36, and MAEBL
(3) **U**pregulated in **I**nfectious **S**porozoites
(4) **S**porozoite-specific proteins
(5) Homologues of *Pf* **L**iver **S**tage **A**ntigens
(6) **S**erial **A**nalysis of **G**ene **E**xpression of **S**alivary **G**land **S**porozoites
(7) All available *Pb* sequences from PLASMODB (http://plasmodb.org/plasmo/)
(8) References in the main text
(9) Analysis was performed starting from Data Set 1. Several sequences, e.g. CSP, were common to different data sets. A sequence was included only once and in the first data set where it was identified.
