# Supplementary material for: Identification of Targets of CD8+ T Cell Responses to Malaria Liver Stages by Genome-wide Epitope Profiling
Source: PLoS Pathog. 2013 May 9;9(5):e1003303. doi: 10.1371/journal.ppat.1003303 (PMC3649980; doi:10.1371/journal.ppat.1003303)
Supplement: Table S2 — Complete list of synthesised peptides. (PDF) [file ppat.1003303.s008.pdf]

**TABLE S2: Peptides and corresponding *P.berghei* target proteins tested**

| Chromosome | Pb gene | Pb protein | Peptide Sequence | Peptide ID |
|---|---|---|---|---|
| I | PBANKA_010060 | SMAC | FNFYNSTFM | 59 |
| | PBANKA_010330 | unknown ORF | FIYAYIEL | 58 |
| | PBANKA_010460 | RAD50 | IIFFFGRL | 247 |
| | PBANKA_010670 | unknown ORF | FSYFFFRL | 346 |
| | PBANKA_010910 | transketolase | SALSNHHVL | 464 |
| | PBANKA_010930 | unknown ORF | INLFYIVL | 67 |
| | | | ISYNSFPL | 71 |
| | | | ISYNSFPLF | 72 |
| | PBANKA_011120 | GTP-binding | RAVVNRTPF | 460 |
| | | | RSLENRNTI | 463 |
| | PBANKA_011240 | unknown ORF | KAYEYVPV | 145 |
| | | | LSYGFKSM | 160 |
| | | | TVIQNFDFL | 230 |
| II | PBANKA_020150 | exported protein | YSMENIQPI | 403 |
| | PBANKA_020310 | PIP5K | VKYTFRSL | 592 |
| | PBANKA_020520 | unknown ORF | SSNTNNHPM | 379 |
| | | | RSIVNLENM | 462 |
| | | | VNLENMDYM | 485 |
| | PBANKA_020580 | UIS1/IK2 | LHYLWWSYL | 85 |
| | | | SNYEKKVLL | 105 |
| | | | VMIKFYTRI | 190 |
| | | | YNYSYKNI | 199 |
| | PBANKA_020620 | unknown ORF | IIWSYAFL | 257 |
| | PBANKA_020870 | LytB | SQIRNTDAI | 373 |
| | PBANKA_020900 | unknown ORF | VSVINQYQL | 113 |
| | | | FLSENQIGYCL | 6 |
| | | | LSYIYFLI | 28 |
| | | | VLFFFFFIV | 48 |
| | | | INTVYNNV | 69 |
| | | | SVINQYQLM | 108 |
| | PBANKA_020910 | TRSP | FSVVNIIIL | 8 |
| | PBANKA_020970 | zinc peptidase | LAITNYDKL | 215 |
| | | | LNYNFRLI | 158 |
| | | | TNYIFIII | 185 |
| | | | YPYTYSYL | 200 |
| | | | NSIKNIEDI | 222 |
| | PBANKA_021060 | Ubi hydrolase | KVYDKYSYI | 83 |
| | | | DTYRYLPL | 57 |
| | | | IFYEYSLI | 62 |
| | | | LIYPYPPL | 87 |
| | | | NQISNFSEI | 94 |
| | | | SIYNMESHL | 101 |
| | | | SSIEFETL | 106 |
| | | | SSISYNNI | 107 |
| | PBANKA_021130 | NFS | INRYFGNM | 68 |
| | | | MTYIYGNA | 91 |
| | PBANKA_021440 | ApiA2 | STYYFKYTL | 181 |
| III | PBANKA_030360 | 26S proteasome | VAILNIALI | 479 |
| | PBANKA_030380 | SufE | TVYIYPKV | 186 |
| | | | VNPNFISL | 191 |
| | PBANKA_030400 | unknown ORF | ISIVNMFYV | 354 |
| | PBANKA_030830 | GAF-domain | KIYQYCTL | 147 |
| | PBANKA_030920 | unknown ORF | KSILNYNTI | 358 |
| | PBANKA_030940 | unknown ORF | MSIFNDQPI | 450 |
| | | | NSIKNYYLM | 455 |
| | PBANKA_031000 | HSP40 | KAYRKMAMM | 77 |
| | | | NTYVYSGV | 95 |
| | PBANKA_031100 | SEC31 | GSLNNYTYL | 423 |
| | | | ASSTNMDTI | 410 |
| | PBANKA_031120 | unknown ORF | YMLKNFNPL | 392 |
| | PBANKA_031180 | AcylCoA synthase | HNLINDIYI | 539 |

**TABLE S2: Peptides and corresponding *P.berghei* target proteins tested (contin.)**

| Chromosome | Pb gene | Pb protein | Peptide Sequence | Peptide ID |
|---|---|---|---|---|
| III | PBANKA_031180 | AcylCoA synthase | VHFSYLPL | 591 |
| | PBANKA_031310 | VPS45 | YSYTNYDAL | 408 |
| | PBANKA_031320 | unknown ORF | IIYPFKNL | 267 |
| | PBANKA_031430 | unknown ORF | CALVNSLYI | 324 |
| | PBANKA_031470 | replication C2 | VSYIFKAL | 488 |
| IV | PBANKA_040100 | unknown ORF | NSIINDDPI | 364 |
| | PBANKA_040120 | ABC transporter | LAYSYYNI | 154 |
| | PBANKA_040200 | unknown ORF | INYAFALL | 274 |
| | PBANKA_040380 | unknown ORF | VIYFFPFL | 307 |
| | | | VIYFYNNL | 308 |
| | PBANKA_040480 | FAD-G3PDH | VSYKYSPI | 489 |
| | PBANKA_040550 | ribosomal L7 | VTYGYPSL | 490 |
| | PBANKA_040610 | unknown ORF | AMIENEEMI | 202 |
| | | | LMHTNMDSI | 216 |
| | | | SALKNVDLL | 227 |
| | PBANKA_040640 | unknown ORF | NMLTNFDFI | 363 |
| | PBANKA_040650 | TCP-1/CPN60 | RSICNKEQI | 461 |
| | PBANKA_040920 | Val tRNA ligase | TSYCNAIYM | 477 |
| | PBANKA_040990 | unknown ORF | ISYLFSYM | 292 |
| | PBANKA_041010 | RING $Zn^{2+}$ finger | SANNNWEYM | 370 |
| | PBANKA_041200 | DER1 | KAWFNLDYI | 357 |
| | PBANKA_041290 | CTRP | FALRNRDYI | 329 |
| | PBANKA_041380 | unknown ORF | LIYSYYNNF | 559 |
| | PBANKA_041410 | $P_i$ pyrophospatase | FQINNKHYM | 421 |
| | | | ISNMFSNL | 439 |
| | PBANKA_041610 | dynein, h chain | IQYNFSPL | 278 |
| | | | SALSNVDIL | 369 |
| | | | IFFRFISM | 429 |
| | | | ISFFLNPL | 436 |
| | PBANKA_041660 | replication A1 | ISIRNWNDM | 438 |
| | PBANKA_041800 | DEAD helicase | FVFIYYMHF | 60 |
| | | | LYYMYYLL | 561 |
| | | | NNLINYSII | 572 |
| | | | QNIRNTDIL | 574 |
| | | | SSNNNYSEM | 584 |
| | PBANKA_041810 | unknown ORF | ISYDYAYL | 290 |
| | | | CAIHNTIYI | 411 |
| | | | SSYVNSYEV | 472 |
| | | | VNYLYYNV | 486 |
| V | PBANKA_050280 | unknown ORF | IFYTYGGV | 542 |
| | | | IIFTFGSF | 544 |
| | | | LIFHYMHL | 558 |
| | | | MKYKYNNL | 562 |
| | PBANKA_050620 | unknown ORF | SSFSFALL | 301 |
| | | | INICNKFFI | 504 |
| | PBANKA_050730 | dynein, h chain | IQIINMDAI | 435 |
| | | | YIYFYKNL | 498 |
| | PBANKA_051090 | ribosomal S2B | SAYANIPVI | 466 |
| | PBANKA_051300 | unknown ORF | SAYTRELPL | 173 |
| | PBANKA_051410 | PRP22 | YSLENLYYL | 395 |
| | PBANKA_051430 | unknown ORF | VSYEYKST | 195 |
| | PBANKA_051640 | unknown ORF | ISFSFGLL | 285 |
| | PBANKA_051830 | unknown ORF | IIFFFNNL | 248 |
| | PBANKA_051870 | Met tRNA ligase | VAIMNIKPL | 480 |
| | PBANKA_051980 | AcylCoA transp. | FAIFNPFFI | 325 |
| | PBANKA_051990 | unknown ORF | FNYSFTNL | 420 |
| | PBANKA_052000 | pyruvate kinase2 | SAVKNIDNI | 465 |

**TABLE S2: Peptides and corresponding *P.berghei* target proteins tested (contin.)**

| Chromosome | Pb gene | Pb protein | Peptide Sequence | Peptide ID |
|---|---|---|---|---|
| VI | PBANKA_060150 | unknown ORF | YSNRNYVYI | 405 |
| | PBANKA_060200 | ORC1 | NIFNFSNL | 92 |
| | PBANKA_060430 | unknown ORF | YSYVFIRL | 499 |
| | PBANKA_060490 | unknown ORF | FTFFYTKL | 121 |
| | | | INYSFEKL | 137 |
| | PBANKA_060530 | TIF5 | YMIKNPPQI | 232 |
| | PBANKA_060590 | unknown ORF | VVFEKDTFL | 53 |
| | PBANKA_060660 | unknown ORF | VIYTYLYL | 310 |
| | PBANKA_060950 | kinesin | ISLNYYNE | 19 |
| | | | ISLNYYNEL | 20 |
| | | | SNFKFAGL | 35 |
| | | | SNFKFAGLM | 36 |
| | | | ISHVFINEM | 70 |
| | PBANKA_060970 | unknown ORF | FMNMNSEPM | 336 |
| | PBANKA_060990 | PFj4 | ISYLYLYF | 139 |
| | PBANKA_061160 | RNDP reductase | VAVCNLASI | 481 |
| | PBANKA_061210 | unknown ORF | IFYYFSFL | 243 |
| | PBANKA_061460 | transporter | ISFIFLFL | 281 |
| | PBANKA_061480 | unknown ORF | YSIKNKTYM | 393 |
| | PBANKA_061570 | dynein, h-chain | FMIENSHIL | 418 |
| | | | IFYEYKYL | 430 |
| | | | SMLYNIVFI | 467 |
| | | | SSNINFDFL | 470 |
| | PBANKA_061860 | unknown ORF | SNISNFDEI | 469 |
| | PBANKA_061890 | unknown ORF | TSVTNAFKI | 111 |
| | PBANKA_062220 | unknown ORF | LSFQFFNL | 295 |
| VII | PBANKA_070190 | GAMA | VSYALFAL | 194 |
| | | | KSLVNGQLI | 212 |
| | PBANKA_070790 | unknown ORF | IIFFFISL | 351 |
| | PBANKA_070800 | SEC23 | VMYSNIEYI | 483 |
| | | | VSLANRAVM | 487 |
| | PBANKA_070880 | ribosomal L4 | FASINDDPI | 331 |
| | PBANKA_071420 | ClpB | ISYRFLPD | 442 |
| | PBANKA_071570 | Lys decarboxylase | TSILNFDML | 476 |
| | PBANKA_071670 | GTP-binding | VIYVFDPL | 311 |
| | PBANKA_071950 | unknown ORF | INYFFNFL | 275 |
| | | | KSISNVTYI | 359 |
| | PBANKA_072030 | proteasome sub. | DAIQNLQYI | 413 |
| | PBANKA_072090 | unknown ORF | IIFLFFFL | 251 |
| VIII | PBANKA_080030 | unknown ORF | INYVYENL | 277 |
| | PBANKA_080230 | Ubi transferase | YMLFNYMPM | 391 |
| | PBANKA_080410 | unknown ORF | MNYYYYNL | 298 |
| | PBANKA_080460 | AAA ATPase | SYYSNFDYI | 473 |
| | PBANKA_080590 | kinesin | IAMLNNTTI | 349 |
| | PBANKA_080810 | unknown ORF | IGWSFNSL | 125 |
| | PBANKA_081030 | unknown ORF | TVYIFSEI | 590 |
| | PBANKA_081110 | helicase | MAYLYSNL | 296 |
| | PBANKA_081130 | Ran-binding | FAYLNCLEL | 415 |
| | PBANKA_081300 | falstatin | IKYLYIFL | 503 |
| | | | IVYSYYRPF | 507 |
| | | | KSPSNFTII | 510 |
| | | | TSYNSFNHL | 521 |
| | | | YLYIFLNL | 526 |
| | PBANKA_081310 | unknown ORF | IIYMYIREF | 502 |
| | | | INIDYENI | 505 |
| | | | MNMKYMNI | 513 |
| | | | MYINYYNL | 514 |
| | | | MYINYYNLM | 515 |
| | | | NMYINYYNL | 516 |
| | | | SGLRNLDNI | 518 |
| | | | SSYCYSKT | 520 |

**TABLE S2: Peptides and corresponding *P.berghei* target proteins tested (contin.)**

| Chromosome | Pb gene | Pb protein | Peptide Sequence | Peptide ID |
|---|---|---|---|---|
| VIII | PBANKA_081320 | unknown ORF | IIYSYCYL | 269 |
| | PBANKA_081340 | unknown ORF | SSNVNQENI | 471 |
| | PBANKA_081390 | proteasoe subun. | FMVKNNDIM | 419 |
| | PBANKA_081410 | unknown ORF | VNSNYIYL | 524 |
| | PBANKA_081630 | unknown ORF | ILYSYSKV | 134 |
| | | | IMYYYAKL | 135 |
| | | | QTYRYMCL | 169 |
| | PBANKA_081970 | DHU synthase | YSLVNAFEL | 401 |
| | PBANKA_082000 | DNAJ chaperone | VIFVYMWL | 428 |
| | PBANKA_082030 | PDI | ITYNFENV | 21 |
| | | | IVFFFTPEL | 22 |
| | | | IVYLASFIL | 23 |
| | | | TSFKMYPSI | 42 |
| | | | YIYIFFIIV | 54 |
| | PBANKA_082040 | sugar transport | MICSFCNL | 163 |
| | | | VSMIFMFI | 193 |
| | PBANKA_082150 | unknown ORF | ISFSYFKL | 286 |
| | PBANKA_082300 | Ubi-like | SIYVFFNL | 300 |
| | PBANKA_082340 | PGK | MAYTFKRV | 89 |
| | PBANKA_082420 | PPLP3 | FAMCNDDSI | 330 |
| | PBANKA_082590 | unknown ORF | FTYSFEVL | 122 |
| | | | IAFYLFYLF | 124 |
| | | | ISYSLMYI | 140 |
| | | | VLFIFYTQL | 189 |
| | PBANKA_082690 | kinase | ACLSNATTI | 533 |
| | | | INFLHTPI | 548 |
| | | | MSSIYSNL | 567 |
| | | | NSTSNMETL | 573 |
| | | | SVYNIYPPL | 586 |
| | PBANKA_082760 | unknown ORF | SSFSNDTTI | 374 |
| | PBANKA_083100 | MSP1 | FSFVFFAI | 537 |
| | | | IGLLFSFV | 543 |
| | | | ISYVSGGL | 554 |
| | | | NALKNNDML | 568 |
| | | | YVIRNPYQL | 600 |
| | PBANKA_083120 | DAG kinase | NMLLNFLPI | 362 |
| | PBANKA_083410 | unknown ORF | IIFTFYLL | 254 |
| IX | PBANKA_090130 | MAEBL | KSLINKEYI | 80 |
| | | | NYYSFTNL | 96 |
| | PBANKA_090210 | SLARP | AIYNCYKNL | 1 |
| | | | ILFSFFSYF | 14 |
| | | | INYYKAQQY | 18 |
| | | | TNYFYTSKM | 40 |
| | | | TNYLTRNSL | 41 |
| | | | NSINNSDTM | 456 |
| | PBANKA_090230 | unknown ORF | KIFVYSNL | 555 |
| | | | YNTQNSMPM | 598 |
| | PBANKA_090240 | unknown ORF | MLYLFGKV | 563 |
| | PBANKA_090315 | unknown ORF | FSYAYFNL | 236 |
| | PBANKA_090460 | unknown ORF | INFIFLII | 546 |
| | | | KSSRFYNL | 556 |
| | PBANKA_090670 | unknown ORF | ISFILYIL | 553 |
| | | | YILSFIPL | 596 |
| | | | YSFIFNLI | 599 |
| | PBANKA_091020 | phophatase 2C | ISYNFYRI | 441 |
| | PBANKA_091030 | GCalpha | MVFAFRYL | 451 |
| | PBANKA_091160 | ribosomal S9 | RALLNACPL | 366 |
| | PBANKA_091230 | EBNA2 binding | YAYINDYTI | 389 |
| | PBANKA_091260 | unknown ORF | FMNTNSKSM | 535 |
| | | | FNLAYNNL | 536 |

**TABLE S2: Peptides and corresponding *P.berghei* target proteins tested (contin.)**

| Chromosome | Pb gene | Pb protein | Peptide Sequence | Peptide ID |
|---|---|---|---|---|
| IX | PBANKA_091260 | unknown ORF | INIKNIIPI | 549 |
| | | | MSLNNHDNI | 566 |
| | | | NIYVYANL | 569 |
| | PBANKA_091380 | RNA polymerase | KNYEYEFL | 148 |
| | | | KSYIYNKI | 152 |
| | | | QIYLRYKEM | 167 |
| | | | KSVINYCVL | 213 |
| | PBANKA_091430 | PDI-related | IAIKNEDII | 425 |
| | PBANKA_091440 | UIS24/HSP70 | NAIINISAI | 452 |
| | PBANKA_091480 | unknown ORF | ISYIFEYL | 291 |
| | PBANKA_091520 | unknown ORF | KMLINWNYV | 445 |
| | PBANKA_091530 | unknown ORF | VSFFFSLM | 593 |
| | PBANKA_091640 | UBI-like | RAISNIEAI | 459 |
| | PBANKA_092430 | ER oxidoreductin | ISYVVPIL | 141 |
| | | | LVYLLYILL | 161 |
| | | | LYFTFAIL | 162 |
| | | | KSIWNRIYL | 210 |
| | PBANKA_092520 | CDPK7 | AMLSNIPYI | 320 |
| | PBANKA_092540 | dynein, h-chain | YAIFNICPN | 493 |
| | PBANKA_092650 | M16 peptidase | INVSNFLEI | 550 |
| | | | NMHKNWNYI | 570 |
| | PBANKA_092910 | AcylCoa-binding | KSFCFFNI | 150 |
| | PBANKA_093030 | GTP-binding | SNYNFEKPF | 177 |
| | PBANKA_093140 | STT3 subunit | ISFIFSVL | 282 |
| | PBANKA_093190 | Ser esterase | IGYVYDLI | 126 |
| | PBANKA_093260 | unknown ORF | INFFFYYL | 272 |
| | PBANKA_093650 | RFC5 | YAPRNLDEL | 494 |
| | PBANKA_093810 | unknown ORF | YMINNDSYI | 390 |
| | PBANKA_093960 | PABP-interacting | SMHINGIHM | 582 |
| X | PBANKA_100250 | unknown ORF | DIYNYYVSL | 4 |
| | PBANKA_100620 | SIAP-1 | YQVINPVPI | 114 |
| | | | YSFGKYTYM | 115 |
| | | | SAFVNYKDI | 577 |
| | PBANKA_100630 | SPECT2 | AVLYYTRL | 3 |
| | | | KNYMYLVCV | 26 |
| | | | VSYLYIEC | 52 |
| | PBANKA_100740 | unknown ORF | LAYIFKQKL | 153 |
| | PBANKA_100750 | ALP | ISFIYNSL | 283 |
| | PBANKA_100950 | Glu synthetase | YSLVNAHEL | 402 |
| | | | IALLNCTPV | 426 |
| | PBANKA_101050 | HOP | YAIENRYNF | 492 |
| | PBANKA_101060 | calmodulin | ISYLFVYL | 293 |
| | PBANKA_101350 | GTPase | ISVLNKDPI | 355 |
| | | | KAIQNSDEI | 256 |
| | PBANKA_101530 | rRNA methyl tf | SSICNSSFI | 377 |
| | PBANKA_101640 | transporter | IIYEFAYL | 258 |
| | PBANKA_101750 | UIS28 | ENYKKYKKL | 5 |
| | | | HYFFYFVFL | 11 |
| | | | IIFGFGFS | 12 |
| | | | IMFGCSSYL | 15 |
| | | | IMFLFMKYF | 16 |
| | | | LNFTFTIMF | 27 |
| | | | THYFFYFVF | 39 |
| | | | LNYDFGNI | 88 |
| | | | QTWYFCYL | 98 |
| | | | RSIMFLFM | 99 |
| | | | ASMANMSSL | 500 |
| | | | FSHKNVMFM | 501 |

**TABLE S2: Peptides and corresponding *P.berghei* target proteins tested (contin.)**

| Chromosome | Pb gene | Pb protein | Peptide Sequence | Peptide ID |
|---|---|---|---|---|
| X | PBANKA_101760 | unknown ORF | YSYLNDVPI | 407 |
| | PBANKA_101800 | unknown ORF | FAVFNILPI | 332 |
| | PBANKA_101950 | ribosomal L5 | TIFYFGHL | 474 |
| | PBANKA_102010 | phosphatase | ISMVYKIL | 138 |
| | PBANKA_102020 | CYP52 | KVIIYTSL | 82 |
| | PBANKA_102050 | unknown ORF | SSICNDDVL | 375 |
| | PBANKA_102430 | RNA helicase | INYLYTFL | 276 |
| | PBANKA_102460 | LISP1 | IALRNINYI | 427 |
| | PBANKA_102790 | SNRPB | IALNNYVPM | 348 |
| | PBANKA_103060 | unknown ORF | RALMNTYPL | 367 |
| | PBANKA_103100 | unknown ORF | VSFSFQNM | 383 |
| | PBANKA_103160 | unknown ORF | IVFLCYPSL | 74 |
| | PBANKA_103380 | unknown ORF | IIYRYFSL | 268 |
| | PBANKA_103390 | ribosomal S8e | RNYKYRAI | 170 |
| | PBANKA_103910 | unknown ORF | IIYLFYFL | 264 |
| XI | PBANKA_110650 | ROM4 | ILYGFATI | 133 |
| | | | SAYRSYTPM | 172 |
| | | | SMYLHGGL | 176 |
| | PBANKA_110770 | SET-domain | IIIKYLMI | 129 |
| | | | IIWQYSPF | 131 |
| | | | SGIMYYTV | 174 |
| | | | SNYYYTLL | 178 |
| | | | SSLIYSYI | 179 |
| | | | SVYSYYNV | 183 |
| | | | ASIWNSYTI | 204 |
| | | | KSFKNIYYL | 209 |
| | PBANKA_111380 | UIS26 | ILQFFANM | 65 |
| | PBANKA_111400 | Asn tRNA ligase | IIYIYNFL | 243 |
| | PBANKA_111490 | PI3K | RAFTNLSEI | 576 |
| | | | SIYLYWFL | 579 |
| | | | SIYLYWFLL | 580 |
| | | | SSFFYNFL | 583 |
| | PBANKA_111510 | Aa transporter | IIFAFIYL | 244 |
| | PBANKA_111590 | unknown ORF | ISFFFSVL | 279 |
| | PBANKA_111600 | TLP | SISSYSHL | 175 |
| | | | KSVNNLDEI | 214 |
| | PBANKA_111830 | unknown ORF | IIFKYPNL | 250 |
| | PBANKA_112060 | unknown ORF | FMLINFLYI | 334 |
| | PBANKA_112370 | unknown ORF | KNLTFSNL | 25 |
| | PBANKA_112680 | HECT-E3 | KAFLYQPV | 144 |
| | | | LSFIFSLL | 159 |
| | | | YAIKNCTAI | 386 |
| | PBANKA_112690 | PK4 | YSLPNMHYI | 400 |
| | PBANKA_112790 | RAP protein | FSLKNKDAM | 342 |
| | PBANKA_112810 | phospholipase | YTLSNWYEL | 531 |
| | PBANKA_112870 | PI/PC transfer | RTYRFIDL | 171 |
| | PBANKA_113000 | Ran-BP | IANLNICFI | 541 |
| | | | MSLANGYYI | 565 |
| | PBANKA_113160 | MCM subunit | SMPRNMDVI | 468 |
| | PBANKA_113280 | unknown ORF | YSILNYFPL | 394 |
| | PBANKA_113530 | unknown ORF | ISFGYSNL | 280 |
| | PBANKA_113670 | GCbeta | AAPKNVLKL | 532 |
| | | | IAFLFADI | 540 |
| | | | ILFIYINI | 545 |
| | | | LAYNFESPI | 557 |
| | | | LSLCNVIEL | 560 |
| | | | TNYTYFYI | 588 |
| | | | TSLDNLEYI | 589 |
| | | | VSILFIYI | 594 |
| | | | VSNKNSDFV | 595 |
| | PBANKA_113730 | unknown ORF | NMLQSYDTI | 219 |

**TABLE S2: Peptides and corresponding *P.berghei* target proteins tested (contin.)**

| Chromosome | Pb gene | Pb protein | Peptide Sequence | Peptide ID |
|---|---|---|---|---|
| XI | PBANKA_113840 | calpain | YSLKNYSTI | 397 |
| | PBANKA_113950 | unknown ORF | IFYSYKNL | 350 |
| | PBANKA_114390 | unknown ORF | IIFIFFLL | 249 |
| | PBANKA_114570 | U2 ribonucleo A | QIFAYAYL | 458 |
| XII | PBANKA_120630 | UIS11 | GTYIFNYL | 61 |
| | | | IIYDFLNL | 64 |
| | | | KTNMNIYPM | 511 |
| | | | YMFQNNQFL | 527 |
| | | | YMVANNYNL | 528 |
| | | | YTLQNYLKI | 530 |
| | PBANKA_120680 | $Zn^{2+}$ finger | SMMYNFKLL | 34 |
| | | | SYFDIFPTM | 38 |
| | | | MMYNFKLL | 90 |
| | | | TMHMNDYPV | 109 |
| | PBANKA_120830 | unknown ORF | KSLSFYSFL | 151 |
| | | | NSIINYKMI | 221 |
| | | | YAHINYNNI | 231 |
| | PBANKA_121100 | GMP5 | YFIENKIPI | 496 |
| | PBANKA_121250 | unknown ORF | ISIDYFIFL | 506 |
| | | | KNYVFNML | 509 |
| | | | VTPEYINV | 525 |
| | PBANKA_121270 | unknown ORF | NQTMNFTPL | 220 |
| | PBANKA_121360 | Cys tRNA synth. | FAIINMITM | 326 |
| | | | KSLKNFITI | 447 |
| | PBANKA_121670 | unknown ORF | FSLLNFEEI | 343 |
| | PBANKA_121760 | H2A.Z | KALMNKVPV | 444 |
| | PBANKA_122100 | unknown ORF | SNFRMISL | 519 |
| | | | YSINNNDLI | 529 |
| | PBANKA_122480 | unknown ORF | SSICNKYVI | 376 |
| | PBANKA_122770 | inositol P-ase | IFFQFFFL | 240 |
| | PBANKA_122790 | unknown ORF | FAIYNTPEM | 328 |
| | PBANKA_122830 | unknown ORF | IYYMRMNEL | 143 |
| | PBANKA_122890 | WARP | ITYIFFSL | 294 |
| | PBANKA_123100 | ribosomal S14 | FASFNDTFI | 206 |
| | PBANKA_123350 | PUF1 | NMIRNIDNI | 218 |
| | | | NSLKNSIII | 224 |
| | | | SNISNDDYL | 228 |
| | PBANKA_123400 | vacuolar ATPase | ASSIFSNL | 117 |
| | PBANKA_123460 | unknown ORF | AILVYSPL | 116 |
| | | | NAIKNVNII | 217 |
| | PBANKA_123540 | unknown ORF | YSLLNNTYI | 398 |
| | PBANKA_123630 | DEAD helicase | VSFQFKNL | 314 |
| | PBANKA_123780 | MDR1 | FAIVNQEPM | 327 |
| | PBANKA_124180 | MCM3 | NSLINYTLM | 457 |
| | PBANKA_124230 | HRP58 | NMMSNPDLI | 454 |
| | PBANKA_124510 | unknown ORF | TIYSFVNL | 303 |
| XIII | PBANKA_130080 | CRMP4 | INFIFSFL | 273 |
| | PBANKA_130200 | unknown ORF | VSYIYIYL | 316 |
| | PBANKA_130320 | unknown ORF | INFLFFLI | 547 |
| | PBANKA_130530 | FACT-S | ISYEYTNI | 440 |
| | PBANKA_130550 | ACP33 | LIQLYAKL | 155 |
| | PBANKA_130630 | unknown ORF | ISIQNETYI | 353 |
| | PBANKA_130660 | unknown ORF | RSIRNTTYL | 368 |
| | PBANKA_130940 | unknown ORF | SNLQNYQSI | 103 |
| | PBANKA_131160 | unknown ORF | SSIFNDDFM | 378 |
| | PBANKA_131480 | EF2 | NAIRNCDPN | 453 |
| | PBANKA_131690 | unknown ORF | IIYKYFNI | 431 |
| | PBANKA_131740 | chaperone p23 | FAVRNSRAI | 414 |
| | | | FIYFFLRL | 417 |
| | PBANKA_131790 | unknown ORF | FSIVNGQFI | 339 |
| | PBANKA_131820 | NifU | ASHLNLPPV | 203 |

**TABLE S2: Peptides and corresponding *P.berghei* target proteins tested (contin.)**

| Chromosome | Pb gene | Pb protein | Peptide Sequence | Peptide ID |
|---|---|---|---|---|
| XIII | PBANKA_132010 | UIS25 | KSSVFSNL | 81 |
| | PBANKA_132220 | unknown ORF | ASIVNYDTN | 321 |
| | PBANKA_132650 | unknown ORF | FSILNTFLI | 338 |
| | PBANKA_132670 | unknown ORF | YSYVNNTFI | 409 |
| | PBANKA_132680 | replication C3 | TSFRFSPL | 304 |
| | PBANKA_132800 | UIS2 | KNFEWHKIM | 24 |
| | | | RNFFFYYNI | 31 |
| | PBANKA_132970 | 26S proteasome | IKYEYVNL | 434 |
| | PBANKA_133090 | unknown ORF | SSFVYQNL | 302 |
| | PBANKA_133130 | unknown ORF | FSHVNSFYI | 337 |
| | | | ANLIYANL | 534 |
| | | | INYVFKLL | 551 |
| | | | IQYIFICL | 552 |
| | | | SKLRNIDYL | 581 |
| | PBANKA_133330 | unknown ORF | IIFFFFHL | 245 |
| | PBANKA_133440 | anion transport | YSLLNYIPL | 399 |
| | PBANKA_133460 | DAG kinase | FNMTNYFSI | 208 |
| | PBANKA_133510 | unknown ORF | KILKFINV | 146 |
| | | | VSYSSYYL | 196 |
| | PBANKA_133590 | RNA polymerase | QIYGYAIV | 166 |
| | PBANKA_134090 | unknown ORF | TSICNKVPM | 382 |
| | PBANKA_134210 | unknown ORF | YSLFNQTTL | 396 |
| | PBANKA_134250 | unknown ORF | TIQNFSSL | 184 |
| | | | NSYVNTGPL | 225 |
| | | | RSIGNIEFL | 226 |
| | | | STIQNFSSL | 229 |
| | PBANKA_134360 | unknown ORF | VHFKYISL | 187 |
| | PBANKA_134410 | myosin | CSYIYAYA | 119 |
| | | | FATSNSAYL | 207 |
| | PBANKA_134790 | UBA | FSIWNYIYI | 340 |
| | PBANKA_134980 | TRAP/SSP2/S8 | SALLNVDNL | 100 |
| | PBANKA_135240 | unknown ORF | VSYLHSPL | 317 |
| | PBANKA_135400 | DBR1 | SAPQNLDPL | 371 |
| | PBANKA_135520 | aconitase | ANYLASPAL | 2 |
| | | | SAIRNCDNL | 32 |
| | PBANKA_135660 | UDP-N-AG-PP | QNLQNGTII | 575 |
| | | | SIYEHQNL | 578 |
| | | | SSVSNNNTL | 585 |
| | PBANKA_135900 | sec61alpha | IIYQFYEM | 433 |
| | PBANKA_135920 | ALBA2 | SSVVNVAEM | 380 |
| | PBANKA_136260 | ATPase | MSKKYYRL | 164 |
| | | | SSLSYASL | 180 |
| | PBANKA_136430 | glycerol kinase | FGITNMYAL | 416 |
| | PBANKA_136520 | thioredoxin-like | IFFLFLTL | 428 |
| XIV | PBANKA_140070 | unknown ORF | LGITFAIL | 84 |
| | | | TYYHYWIPM | 112 |
| | PBANKA_140080 | UIS3 | FNFPFFNK | 7 |
| | | | TTFFFNPC | 43 |
| | | | TTFFFNPCF | 44 |
| | | | VFFVFYVLY | 46 |
| | | | VFYVLYITT | 47 |
| | | | VLYYMYKSH | 49 |
| | | | NSLKNIDTA | 517 |
| | | | VFFVFYVL | 523 |
| | PBANKA_140290 | unknown ORF | FSYVNGNYM | 347 |
| | PBANKA_140370 | ApiAP2 | YALSNNYFI | 388 |
| | PBANKA_140670 | CP synthetase | IAICNMENI | 424 |
| | PBANKA_140690 | unknown ORF | FSFLFNNL | 235 |
| | | | IIFSFFYL | 253 |
| | PBANKA_141340 | UIS23 | KNFFFKNPV | 78 |

TABLE S2: Peptides and corresponding *P.berghei* target proteins tested (contin.)

| Chromosome | Pb gene | Pb protein | Peptide Sequence | Peptide ID |
|---|---|---|---|---|
| XIV | PBANKA_141380 | unknown ORF | ASNSNYDYI | 323 |
| | PBANKA_141570 | ApiAP2 | GSIANLDSI | 538 |
| | | | MNIPYMNL | 564 |
| | | | NMHNNYMPL | 571 |
| | | | TNYRYENI | 587 |
| | | | YMNNNYLTV | 597 |
| | PBANKA_141830 | rhoptry protein 2 | VNFLYYNV | 484 |
| | PBANKA_141930 | unknown ORF | ISLVFYNL | 288 |
| | PBANKA_141940 | unknown ORF | YALSNLYEI | 387 |
| | PBANKA_142240 | importin beta | VSYFYSYL | 315 |
| | PBANKA_142280 | unknown ORF | VYYIYAPF | 198 |
| | PBANKA_142290 | unknown ORF | INYNHKERF | 17 |
| | | | YSLSNHDEI | 55 |
| | PBANKA_142520 | unknown ORF | QIYKYVFPL | 30 |
| | | | SIFIFYTPV | 33 |
| | | | IIFAWASI | 63 |
| | PBANKA_142690 | helicase | KSIVNDYDM | 446 |
| | PBANKA_142780 | C50 peptidase | AYYYFEKRL | 56 |
| | | | FSMVNYFTI | 345 |
| | | | CIFIFGNL | 412 |
| | | | IIYLHNNL | 432 |
| | | | ISIEYSFL | 437 |
| | | | TSYLYDRL | 478 |
| | PBANKA_142870 | TIF3 subunit 10 | KVYNYISI | 512 |
| | PBANKA_142920 | S20 | VNYSFLYLF | 51 |
| | | | SNVCRFAPL | 104 |
| | PBANKA_143230 | CelTOS | FVFFCFFNV | 9 |
| | | | HTYSLVSPV | 10 |
| | | | SVFVFFCFF | 37 |
| | | | VFFCFFNVL | 45 |
| | | | VNRHTYSLV | 50 |
| | | | VFFCFFNV | 522 |
| | PBANKA_143300 | unknown ORF | FSLLNFYFL | 344 |
| | PBANKA_143380 | unknown ORF | IFYFYYNL | 242 |
| | PBANKA_143420 | Arg tRNA synth. | AVVTYANM | 118 |
| | | | IAALYYRL | 123 |
| | PBANKA_143470 | clathrin h-chain | ITLNYINL | 443 |
| | PBANKA_143820 | DNA gyrase alpha | YAHNNKTYI | 384 |
| | PBANKA_143920 | PABP | YAYVNYHNL | 495 |
| | PBANKA_144070 | UBA | FIYAFANL | 234 |
| | | | YAFANLRAM | 491 |
| | PBANKA_144820 | unknown ORF | FSVKNEPTL | 422 |
| | | | LSLVNADYV | 448 |
| | | | MMILNFDVI | 449 |
| | | | TSFLFRQL | 475 |
| | PBANKA_144870 | unknown ORF | FMLLNYKYI | 335 |
| | PBANKA_144930 | CPW-WPC family | ISFYFMFL | 287 |
| | PBANKA_144960 | unknown ORF | FAHRNMNSL | 205 |
| | | | NSLINIFPI | 223 |
| | | | NSLINIFPI | 365 |
| | PBANKA_145420 | unknown ORF | IMYKKLNVL | 66 |
| | | | ITYVQNNL | 73 |
| | | | IVIKYHHI | 75 |
| | | | KNYRYSVM | 79 |
| | | | LILRFEHL | 86 |
| | | | QIMNYTNL | 97 |
| | | | TNFMFSSV | 110 |
| | PBANKA_145570 | unknown ORF | IIYQYVLL | 352 |
| | PBANKA_146270 | unknown ORF | FSYNYFKM | 120 |

**TABLE S2: Peptides and corresponding *P.berghei* target proteins tested (contin.)**

| Chromosome | Pb gene | Pb protein | Peptide Sequence | Peptide ID |
|---|---|---|---|---|
| XIV | PBANKA_146270 | unknown ORF | IIFKIAGL | 127 |
| | | | INQKFLFL | 136 |
| | | | LNIFYNNL | 157 |
| | PBANKA_146330 | unknown ORF | YTSTNLDYI | 233 |
| unknown | PB000530.00.0 | unknown | KSLENIDEL | 211 |
| | PB000530.00.0 | unknown | VSHGFSEL | 192 |
| | PB100935.00.0 | unknown | IIFYYALL | 256 |
| | - | UIS6 | IYYMHFLNF | 76 |
| | PB100963.00.0 | guanyl cyclase | IIQKYNNV | 130 |
| | | | VIYVYFIL | 188 |
| | PB101556.00.0 | unknown | ISYAYCAL | 289 |
| | PB101774.00.0 | unknown | IIFFFFYL | 246 |
| | PB102211.00.0 | unknown | IKYYFQNEL | 132 |
| | PB102515.00.0 | unknown | ISFPFPRL | 284 |
| | PB102994.00.0 | unknown | IIYTYAYM | 270 |
| | PB103202.00.0 | unknown | SIYKFASL | 372 |
| | PB103347.00.0 | unknown | VSYNFLFL | 319 |
| | PB103980.00.0 | unknown | IVFFFAFL | 142 |
| | PB104119.00.0 | unknown | VFFQFGNL | 305 |
| | PB104445.00.0 | unknown | MSYFFFPL | 299 |
| | PB104461.00.0 | unknown | LIYVYFFV | 156 |
| | | | VVYMSFNSI | 197 |
| | | | YVYFFVNSL | 201 |
| | PB104640.00.0 | unknown | SSYTNMFPL | 381 |
| | PB104885.00.0 | unknown | IIYIFYLL | 262 |
| | PB105090.00.0 | unknown | SVIIYKSL | 182 |
| | PB105459.00.0 | unknown | IFFFFSYL | 239 |
| | PB105607.00.0 | unknown | IIHFFINI | 128 |
| | | | MTLKFIYV | 165 |
| | | | QNYIFFAL | 168 |
| | PB105775.00.0 | unknown | IFFFFFYL | 238 |
| | PB106391.00.0 | unknown | YSVVNYELI | 406 |
| | PB106640.00.0 | unknown | IFFSFFFL | 241 |
| | PB106724.00.0 | unknown | MNFLYANL | 297 |
| | PB107429.00.0 | unknown | IIYGFAFL | 261 |
| | PB107461.00.0 | unknown | SLFVFIYL | 102 |
| | PB107666.00.0 | unknown | FAYSNMTNI | 333 |
| | | | YSMENLIYI | 404 |
| | PB108856.00.0 | unknown | NNYKFSLL | 93 |
| | PB301444.00.0 | unknown | KIFYFGSV | 508 |
| | PB301531.00.1 | unknown | IILLFSLL | 13 |
| | PB400289.00.0 | unknown | VFYVFFNL | 306 |
| | PB400627.00.0 | unknown | VSYLYVLL | 318 |
| | PB401053.00.0 | unknown | VNYHYALL | 313 |
| | PB401186.00.0 | unknown | NMLINRYYM | 361 |
| | PB401464.00.0 | unknown | IMFRFAFL | 271 |
| | PB401772.00.0 | unknown | VIYYFIPL | 312 |
| | PB401949.00.0 | unknown | VIYLFNFL | 309 |
| | PB402277.00.0 | unknown | IIYFYLFL | 260 |
| | | | IIYLYLFL | 265 |
| | PB402615.00.0 | unknown | KNYYFSYV | 149 |
| | PB403128.00.0 | unknown | YAIINRVNM | 385 |
| | PB403225.00.0 | unknown | IIFNFAQL | 252 |
| | PB403477.00.0 | unknown | IIYFFLNL | 259 |
| | PB404783.00.0 | unknown | ASMVNMLHM | 322 |
| | PB405175.00.0 | unknown | FSLINTFVI | 341 |
| | PB405410.00.0 | unknown | KSLINMYFI | 360 |
| | PB405453.00.0 | unknown | IIFYFIPL | 255 |
| | PB405552.00.0 | unknown | IFFFFFFL | 237 |
| | PB405815.00.0 | unknown | IIYMFLFL | 266 |
