# Supplementary material for: Identification of Targets of CD8+ T Cell Responses to Malaria Liver Stages by Genome-wide Epitope Profiling
Source: PLoS Pathog. 2013 May 9;9(5):e1003303. doi: 10.1371/journal.ppat.1003303 (PMC3649980; doi:10.1371/journal.ppat.1003303)
Supplement: Table S3 — Tolerisation and multiple immunisation with Pb γ-Spz. (DOCX) [file ppat.1003303.s009.docx]

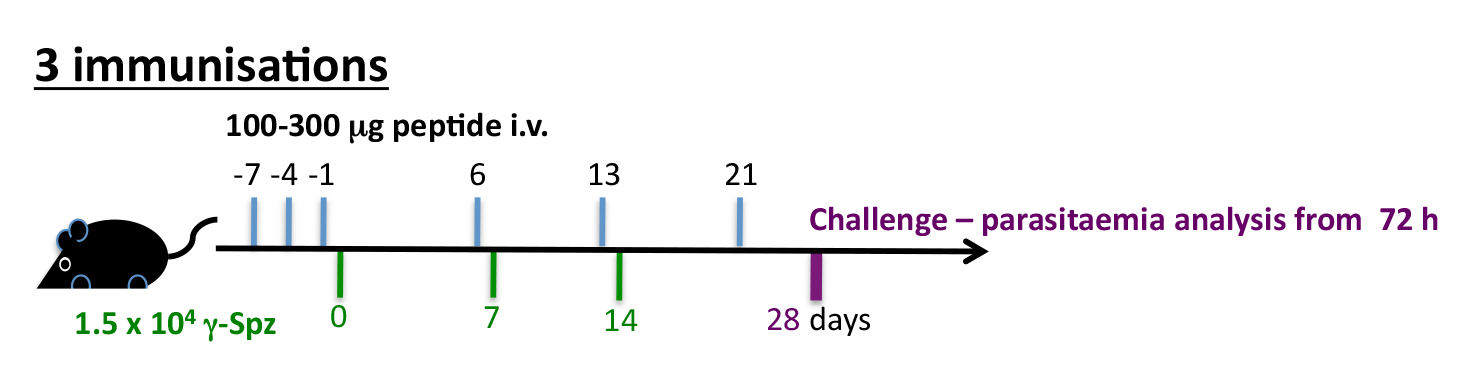


|  | No. protected/  No. challenged  (%) | Prepatency  (in days) |
| --- | --- | --- |
| Sham tolerised | 12/12  (100) | - |
| *Pb*S20_318_ tolerised | 6/6  (100) | - |
| *Pb*TRAP_130_ tolerised | 12/12  (100) | - |
| Naïve challenged | 0/9  (0) | 3 |

**Table S3**
